# I Don't Have a Diagnosis for You: Preparing Medical Students to Communicate Diagnostic Uncertainty in the Emergency Department

**DOI:** 10.15766/mep_2374-8265.11218

**Published:** 2022-02-04

**Authors:** Maria Poluch, Jordan Feingold-Link, Nethra Ankam, Jared Kilpatrick, Kenzie Cameron, Shruti Chandra, Amanda Doty, Matthew Klein, Danielle McCarthy, Kristin Rising, David Salzman, Deborah Ziring, Dimitrios Papanagnou

**Affiliations:** 1 Medical Student, Sidney Kimmel Medical College at Thomas Jefferson University; 2 Associate Professor, Department of Rehabilitation Medicine, Sidney Kimmel Medical College at Thomas Jefferson University; 3 Medical Education Fellow, Department of Emergency Medicine, Sidney Kimmel Medical College at Thomas Jefferson University; 4 Research Professor, Division of General Internal Medicine and Geriatrics, Department of Medicine and Department of Medical Education, Northwestern University Feinberg School of Medicine; 5 Associate Professor, Department of Emergency Medicine, Sidney Kimmel Medical College at Thomas Jefferson University; 6 Research Coordinator, Department of Emergency Medicine, Thomas Jefferson University; 7 Assistant Professor, Department of Emergency Medicine, Northwestern University Feinberg School of Medicine; 8 Associate Professor, Department of Emergency Medicine, Northwestern University Feinberg School of Medicine; 9 Associate Professor and Director of Acute Care Transitions, Department of Emergency Medicine, Sidney Kimmel Medical College at Thomas Jefferson University; 10 Associate Professor, Department of Emergency Medicine and Department of Medical Education, Northwestern University Feinberg School of Medicine; 11 Clinical Associate Professor, Department of Medicine, and Senior Associate Dean for Academic Affairs, Sidney Kimmel Medical College at Thomas Jefferson University; 12 Professor and Vice Chair for Education, Department of Emergency Medicine, Sidney Kimmel Medical College at Thomas Jefferson University; Macy Faculty Scholar, Josiah Macy Jr. Foundation

**Keywords:** Diagnostic Uncertainty, Communication Skills, Role-Play, Flipped Classroom, Virtual Learning

## Abstract

**Introduction:**

Diagnostic uncertainty abounds in medicine, and communication of that uncertainty is critical to the delivery of high-quality patient care. While there has been training in communicating diagnostic uncertainty directed towards residents, a gap remains in preparing medical students to understand and communicate diagnostic uncertainty. We developed a session to introduce medical students to diagnostic uncertainty and to practice communicating uncertainty using a checklist during role-play patient conversations.

**Methods:**

This virtual session was conducted for third-year medical students at the conclusion of their core clerkships. It consisted of prework, didactic lecture, peer role-play, and debriefing. The prework included reflection prompts and an interactive online module. The role-play featured a patient complaining of abdominal pain being discharged from the emergency department without a confirmed diagnosis. Students participated in the role of patient, provider, or observer.

**Results:**

Data from an anonymous postsession survey (76% response rate; 202 of 265 students) indicated that most students (82%; 152 of 185) felt more comfortable communicating diagnostic uncertainty after the session. A majority (83%; 166 of 201) indicated the session was useful, and most (81%; 149 of 184) indicated it should be included in the curriculum.

**Discussion:**

This virtual session requires few facilitators; has peer role-play, eliminating the need for standardized patients; and is adaptable for in-person teaching. As its goal was to introduce an approach to communicating diagnostic uncertainty, not achieve mastery, students were not individually assessed for proficiency using the Uncertainty Communication Checklist. Students felt the session intervention was valuable.

## Educational Objectives

By the end of this activity, learners will be able to:

1. Define diagnostic uncertainty.
2. Discuss the impact diagnostic uncertainty has on patient care.
3. Explain the importance of effectively communicating diagnostic uncertainty to patients.
4. Describe key steps in communicating diagnostic uncertainty.
5. Participate in a conversation where diagnostic uncertainty is communicated to a patient at the point of discharge.

## Introduction

Effective communication skills are critical to providing high-quality care to patients.^1^ Consequently, training interventions aimed at developing these skills in medical trainees are common,^1^ but patient-physician communication often requires clinicians to navigate situations complicated by uncertainty.^2^

Diagnostic uncertainty is an innate part of medicine and has been defined as “the subjective perception of an inability to provide an accurate explanation of the patient's health problem.”^3^ The way in which this particular form of uncertainty is communicated to patients has implications for the patient's perception of physician competence and visit satisfaction.^4^ A survey conducted in 2015 revealed that 37% of patients were discharged from the emergency department (ED) with an uncertain diagnosis.^5^ In addition, the way in which providers respond to uncertainty impacts patient care.^6–8^ In a study conducted on internal medicine residents, investigators found that resident uncertainty results in delays of care and patient harm.^7^ Dr. Kristin Rising and colleagues found that nearly half of surveyed emergency medicine residents found difficulty with conversations involving diagnostic uncertainty and that 51% had a strong desire for additional training. Of participants, 62% reported that medical school training had prepared them *not at all* or *minimally* for these conversations.^9^ Despite this recognized challenge in communication, most existing interventions aim to improve communication skills^10–13^ or reasoning under uncertainty,^14,15^ but there are few interventions aimed at educating medical students on how to communicate uncertainty to patients or to tolerate uncertainty.

Intolerance of uncertainty, defined as one's tendency to consider the occurrence of negative events as unacceptable independent of the probability of that occurrence, can be measured using the Intolerance of Uncertainty Scale (IUS).^16^ For clinicians, higher scores on the IUS correlate with failure to comply with evidence-based guidelines, increased test ordering,^17^ withholding information from patients and families,^18^ and higher likelihood of burnout.^19,20^ Increasing self-compassion is one way to cope with the stress associated with reconciling uncertainty encountered in clinical practice, as shown by Martin Delaney's study conducted with nurses.^21^ Self-compassion can be measured using instruments such as the Self-Compassion Scale.^22^ Higher self-compassion scores are correlated with decreased stress, burnout, and depression.^23,24^

To address this, we designed a session for third-year medical students to (1) assess self-compassion, (2) introduce tools to communicate diagnostic uncertainty, and (3) participate in a simulated conversation where diagnostic uncertainty was communicated to a patient using a tool. We focused on the Uncertainty Communication Checklist (UCC),^25^ which was developed to establish an approach for effective discharge communication in the ED. Both the items and the wording of the steps in the UCC were devised with help from patients to create a tool that ensured the best patient experience while also including relevant test results, next steps, and reasons to return to care.^25^ The session featured prework, didactic lecture materials, and a peer role-play case, and was intended for third-year students who had completed their core clerkships and had gained experiences with diagnostic uncertainty to reflect upon. Because of the impact that intolerance of uncertainty and self-compassion have on patient and physician experiences, we added the Self-Compassion Scale as a reflective component to the prework and didactic lecture.

## Methods

To better prepare medical students for the communication scenarios they will encounter as residents and physicians, we developed a flipped classroom session to expose undergraduate medical students to tools for communicating diagnostic uncertainty in an ED discharge encounter setting, including the UCC (Appendix A), a 21-item tool developed to train and assess medical providers in effectively communicating uncertainty.^25^

### Prework and Preparation

Prior to the session, we assigned students several reflection exercises. The prework reflection prompts (Appendix B) allowed students to think about situations in which they had experienced uncertainty during clinical clerkships. They submitted responses electronically through Canvas LMS (Instructure). We also provided students with links to complete two published instruments with validity evidence in order to measure their comfort with uncertainty (IUS, Appendix C) and their self-compassion (Self-Compassion Scale, Appendix D).^16,22^ We aggregated the data from these scales and incorporated them into the initial lecture during the session.

The final prework item was an online module, which represented the core content for preparing for the session (Appendix E). Authors Kristin Rising, Danielle McCarthy, Kenzie Cameron, Amanda Doty, David Salzman, and Dimitrios Papanagnou developed the interactive module through support from the Agency for Healthcare Research and Quality. We created this module using Articulate Rise 360 (Articulate Global) software and designed it to introduce learners to the relevant concepts of uncertainty, as well as the 21 items of the UCC (Appendix A).

The module then introduced the steps needed to effectively communicate diagnostic uncertainty to patients, including a modeled case example and self-testing items to reinforce knowledge. Students signed an attestation to confirm their prework completion prior to the start of the session.

#### Equipment

In general, this session required few materials for implementation. We used a virtual audiovisual platform with capability for breakout rooms. All students and facilitators needed a computer or tablet device with a functional webcam and microphone to be able to access the online meeting and participate in the session.

Given the large number of students participating (the entire third-year class), we planned four separate 90-minute sessions, each designed for simultaneous participation by 60–70 students. We provided students with a link to a virtual, audio-video meeting for each session.

#### Personnel

We recruited facilitators who were either senior residents, fellows, or attending physicians with experience addressing diagnostic uncertainty and moderating small-group discussions. Prior to the session, facilitators were told to review the instructions for each of the student roles, the UCC, and the debriefing questions (Appendix F) in order to be familiar with the material. The debriefing questions in the guide were based on Brookfield's Critical Incident Questionnaire^26^ and were designed to elicit concrete feedback from students concerning their role-play experience.

One instructor presented the initial didactic PowerPoint lecture (Appendix G). A second instructor, who was comfortable working a virtual audiovisual platform, assigned students to breakout rooms during the lecture. An additional four facilitators facilitated the small-group debriefing sessions.

### Implementation

We instructed students to complete an attestation to acknowledge that the required presession work had been completed before attending the session. Once students submitted the attestation, they gained access to the virtual platform for the main session.

We began the session with a 20-minute didactic lecture (Appendix G) to expand upon the materials presented in the prework module, underscore the frequency and importance of effectively communicating diagnostic uncertainty, and set the stage for the peer role-play and debriefing session.

Immediately following the initial lecture, we shared role-play instruction documents (Appendix H) for the three roles (patient, provider, and observer) via the virtual meeting chat function. We instructed students to download the document, accept the breakout room invitation, and self-assign one student to each role. They were told that the role-play should take 10 minutes, with 5 minutes allotted for peer-to-peer feedback from the student in the observer role using the UCC.

The peer role-play exercise features a patient presenting to the ED with abdominal pain and needing to be discharged after a complete evaluation and workup without a diagnosis to explain symptoms. We selected this presentation given the frequency of patients presenting with abdominal pain to the ED without a final clinical diagnosis. The scenario was designed for three learners, with one playing the role of the patient, one playing the role of the provider, and one observing the discharge conversation with the intent to provide peer feedback at the end of the exercise.

To encourage emotional engagement, we provided the student playing the role of the patient with contextual information to affectively understand the perspective of the patient (i.e., the patient had recently learned that a friend had been diagnosed with colon cancer and was now concerned that their abdominal pain might be secondary to an underlying malignancy). We also provided the student playing the physician with pertinent information and findings that could support a discharge conversation (i.e., a computed tomography scan of the abdomen that was interpreted as being free of any pathologic abnormalities). Finally, we provided the student observer with a copy of the UCC to assess the conversation as it took place.

Students completed the role-play and then exited their respective breakout rooms to reenter the main virtual platform room for debriefing.

### Debriefing

Following the role-play, all learners entered one of three virtual platform breakout rooms based on the role that they had played to participate in instructor-led debriefing sessions with 10–12 students. We placed students of the same role together for debriefing to facilitate transit and organization. We held a brief facilitator training in advance of the session to prepare facilitators to have an open discussion about diagnostic uncertainty and share their experiences, while incorporating the specific items on the debriefer guide (Appendix F). As the debrief sessions ended, facilitators directed students to complete postsession evaluations.

### Assessment

After the debriefing, all participants received an anonymous postsession questionnaire. The survey (Appendix I) contained 10 questions regarding the students' experiences with the session and about their learning. As a means of gauging learning, we asked students to indicate three actions discussed during the session that they would apply when communicating with a patient during times of diagnostic uncertainty. Using a Likert scale, we solicited students' self-reported comfort with communicating uncertainty, their rating of the overall usefulness of the session in preparing for diagnostic uncertainty, and their feedback on the prework and the session. We also asked students to provide suggestions to improve the session.

## Results

The session was run with a total of 265 third-year medical students. After the session, most students (76%; 202 of 265) submitted answers to an anonymous postsession evaluation survey. The majority of respondents (88%; 178 of 202) completed every Likert item on the survey. Data for each item were analyzed independently so that students who completed only a portion of the survey would be included.

Survey results showed that 82% of students (152 of 185) felt either *somewhat comfortable* or *extremely comfortable* with communicating diagnostic uncertainty after this session. Most students (83%; 166 of 201) indicated that the session was either *moderately useful*, *very useful*, or *extremely useful* in preparing them to communicate diagnostic uncertainty with a patient.

Survey results revealed the prework module was well received, with 89% of students (162 of 182) indicating that the prework UCC module was either *moderately useful*, *very useful*, or *extremely useful* in preparing them for the session. Additionally, students felt it was useful to reflect on their views of uncertainty before the session, with 81% of students (162 of 199) rating this as *moderately useful*, *very useful*, or *extremely useful*. The majority of students (81%; 149 of 184) felt that session should be included in the formal curriculum moving forward.

As a way to gauge knowledge acquired during the session, students were asked to list three specific actions they would apply in the future when communicating diagnostic uncertainty to their patients. The majority of student respondents (93%; 124 of 132), based on review of the responses by the course directors, did accurately list three specific steps from the UCC.

Learners were also asked to offer suggestions to improve the session in the future. Some key representative quotes from their feedback included the following:

• “The online preparation module was FANTASTIC. It was especially helpful to hear quotes from real patients about how they felt.”
• “I found the modules done beforehand were extremely helpful. I just wish we could have been given more time to prepare with the physician/patient information prior to role playing. It is difficult to scan an encounter on a word document briefly and then have a long, meaningful conversation.”
• “I think it would be beneficial to allow students to participate in more than one role.”

## Discussion

Our uncertainty session was well received by medical students and proved to be a feasible and valuable experience for learning and practicing the skills associated with communicating diagnostic uncertainty. Survey results revealed the prework effectively primed the discussion of the importance of communicating uncertainty and set the stage for student engagement in the role-play encounter. Students felt the online prework reflections and module were valuable. The online prework was designed to allow students to reflect on their prior experience with uncertainty, self-assess their own comfort with uncertainty and a particular skill related to comfort with uncertainty (self-compassion), and then provide a framework for communication of diagnostic uncertainty prior to entering the session. This prework can be scaled down if these topics are addressed elsewhere in a curriculum.

For developing a skill like communicating uncertainty, role-play with a dedicated debriefing is an ideal instructional modality. It affords students the opportunity to gain simulated experience having difficult conversations with patients during times of diagnostic uncertainty while using the checklist for guiding prompts. Importantly, this benefit was not limited to students playing the role of the provider. Students who played the role of the patient and observer also felt more comfortable with communicating diagnostic uncertainty following the session. By completing the role-play in breakout rooms containing only students, the stress of assessment was avoided, and learners were given the opportunity to practice more freely. The facilitator-guided debriefing sessions allowed the students to self-reflect on how they performed during the role-play and to discuss lessons learned with their peers.

### Limitations

Given the amount of curricular prework assigned to students, we decided against a pre-post survey design for assessing student learning. This represented a major limitation in our ability to specifically measure learning among the students who participated. Additionally, we decided not to evaluate students' performance using the UCC during the role-play. The UCC was developed for clinicians working in an ED setting, but this session was designed for all medical students who completed their core clerkships, regardless of their future clinical interest. For this reason, we aimed to more broadly introduce the concepts of uncertainty and the impact that effectively communicating uncertainty has on patient care using the specific example of a patient being discharged from the ED with an uncertain diagnosis to ground the material. This session was focused on the content that the provider should bring into the uncertainty conversations. The methods used to deliver this message, specifically teach-back, empathetic questions, and shared decision-making, are taught in other parts of our curriculum.

### Lessons Learned

After reflecting on our experiences with the session and reviewing participant feedback, we suggest that others implementing this training eliminate the observer role entirely. Instead, participants should be broken down into pairs (i.e., one student plays the role of the patient, and another student plays the role of the physician). Additionally, debrief sessions should include students who played different roles to allow students to hear more varied experiences. Finally, we recommend that more time be allotted for the role-play, with dedicated time to review instructions and truly get into character to fully maximize the patient's affective state.

### Future Adaptations

This session could be easily adopted at other medical schools to introduce medical students to effectively communicating diagnostic uncertainty. One key feature of this session that makes it convenient to use is the lack of standardized patients, which helps to keep the session execution cost low. Another key feature is the session's adaptability to different audience sizes, settings, and roles. Depending on the size of the learner audience, the number of small-group debriefs can be scaled up or down. Similarly, this session was hosted virtually but can easily be adapted to an in-person format. Lastly, residents and other health care professionals, including nurse practitioners, physician assistants, and other personnel who communicate directly with patients about their diagnosis, could benefit from a similar role-play technique using a validated tool to communicate diagnostic uncertainty to patients. Future sessions should include scenarios representing examples of diagnostic uncertainty that are present in many other specialties and clinical learning contexts.

## Appendices


Uncertainty Communication Checklist.docxPrework Reflection Prompts.docxIntolerance of Uncertainty Scale.docxSelf-Compassion Scale Short Form.pdfUncertainty Articulate Module folderDebrief Facilitator Prompts.docxCommunicating Diagnostic Uncertainty Slides.pptxSimulation Student Role-Play Instructions.docxPostsession Survey.docx
*All appendices are peer reviewed as integral parts of the Original Publication.*

## References

[1] Maguire P, Pitceathly C. Key communication skills and how to acquire them. BMJ. 2002;325(7366):697–700. 10.1136/bmj.325.7366.697 12351365 PMC1124224

[2] O'Riordan M, Dahinden A, Aktürk Z, et al. Dealing with uncertainty in general practice: an essential skill for the general practitioner. Qual Prim Care. 2011;19(3):175–181. 21781433

[3] Bhise V, Rajan SS, Sittig DF, Morgan RO, Chaudhary P, Singh H. Defining and measuring diagnostic uncertainty in medicine: a systematic review. J Gen Intern Med. 2018;33(1):103–115. 10.1007/s11606-017-4164-1 28936618 PMC5756158

[4] Bhise V, Meyer AND, Menon S, et al. Patient perspectives on how physicians communicate diagnostic uncertainty: an experimental vignette study. Int J Qual Health Care. 2018;30(1):2–8. 10.1093/intqhc/mzx170 29329438

[5] Wen LS, Espinola JA, Kosowsky JM, Camargo CA Jr. Do emergency department patients receive a pathological diagnosis? A nationally-representative sample. West J Emerg Med. 2015;16(1):50–54. 10.5811/westjem.2014.12.23474 25671008 PMC4307726

[6] Alam R, Cheraghi-Sohi S, Panagioti M, Esmail A, Campbell S, Panagopoulou E. Managing diagnostic uncertainty in primary care: a systematic critical review. BMC Fam Pract. 2017;18:79. 10.1186/s12875-017-0650-0 28784088 PMC5545872

[7] Farnan JM, Johnson JK, Meltzer DO, Humphrey HJ, Arora VM. Resident uncertainty in clinical decision making and impact on patient care: a qualitative study. Qual Saf Health Care. 2008;17(2):122–126. 10.1136/qshc.2007.023184 18385406

[8] Politi MC, Clark MA, Ombao H, Légaré F. The impact of physicians' reactions to uncertainty on patients' decision satisfaction. J Eval Clin Pract. 2011;17(4):575–578. 10.1111/j.1365-2753.2010.01520.x 20695949 PMC2978752

[9] Rising KL, Papanagnou D, McCarthy D, Gentsch A, Powell R. Emergency medicine resident perceptions about the need for increased training in communicating diagnostic uncertainty. Cureus. 2018;10(1):e2088. 10.7759/cureus.2088 29564193 PMC5858850

[10] Cowfer B, McGrath C, Trowbridge A. Teaching pediatric palliative care communication skills to fourth-year medical students through role-play. MedEdPORTAL. 2020;16:10991. 10.15766/mep_2374-8265.10991 33094157 PMC7566222

[11] Talwalkar JS, Fortin AH, Morrison LJ, et al. An advanced communication skills workshop using standardized patients for senior medical students. MedEdPORTAL. 2021;17:11163. 10.15766/mep_2374-8265.11163 34124349 PMC8155077

[12] Mulcare M, Naik N, Greenwald P, et al. Advanced communication and examination skills in telemedicine: a structured simulation-based course for medical students. MedEdPORTAL. 2020;16:11047. 10.15766/mep_2374-8265.11047 33365390 PMC7751329

[13] Ho B, Ishizaki A, Ko A, et al. Kim Lee: problem-based learning (PBL) case for delivering uncertain news. MedEdPORTAL. 2013;9:9525. 10.15766/mep_2374-8265.9525

[14] Willett L. Uncertain times: clinical decision-making in sickness and in health. MedEdPORTAL. 2013;9:9620. 10.15766/mep_2374-8265.9620

[15] Battista A, Konopasky A, Ramani D, et al. Clinical reasoning in the primary care setting: two scenario-based simulations for residents and attendings. MedEdPORTAL. 2018;14:10773. 10.15766/mep_2374-8265.10773 30800973 PMC6346281

[16] Carleton RN, Norton MAPJ, Asmundson GJG. Fearing the unknown: a short version of the Intolerance of Uncertainty Scale. J Anxiety Disord. 2007;21(1):105–117. 10.1016/j.janxdis.2006.03.014 16647833

[17] Ghosh AK. On the challenges of using evidence-based information: the role of clinical uncertainty. J Lab Clin Med. 2004;144(2):60–64. 10.1016/j.lab.2004.05.013 15322499

[18] Geller G, Tambor ES, Chase GA, Holtzman NA. Measuring physicians' tolerance for ambiguity and its relationship to their reported practices regarding genetic testing. Med Care. 1993;31(11):989–1001. 10.1097/00005650-199311000-00002 8231339

[19] Kuhn G, Goldberg R, Compton S. Tolerance for uncertainty, burnout, and satisfaction with the career of emergency medicine. Ann Emerg Med. 2009;54(1):106–113.E6. 10.1016/j.annemergmed.2008.12.019 19201058

[20] Simpkin AL, Khan A, West DC, et al. Stress from uncertainty and resilience among depressed and burned out residents: a cross-sectional study. Acad Pediatr. 2018;18(6):698–704. 10.1016/j.acap.2018.03.002 29524616

[21] Delaney MC. Caring for the caregivers: evaluation of the effect of an eight-week pilot mindful self-compassion (MSC) training program on nurses' compassion fatigue and resilience. PLoS One. 2018;13(11):e0207261. 10.1371/journal.pone.0207261 30462717 PMC6248952

[22] Raes F, Pommier E, Neff KD, Van Gucht D. Construction and factorial validation of a short form of the Self-Compassion Scale. Clin Psychol Psychother. 2011;18(3):250–255. 10.1002/cpp.702 21584907

[23] Dev V, Fernando AT III, Consedine NS. Self-compassion as a stress moderator: a cross-sectional study of 1700 doctors, nurses, and medical students. Mindfulness (N Y). 2020;11(5):1170–1181. 10.1007/s12671-020-01325-6 32435318 PMC7223415

[24] Montero-Marin J, Zubiaga F, Cereceda M, Piva Demarzo MM, Trenc P, Garcia-Campayo J. Burnout subtypes and absence of self-compassion in primary healthcare professionals: a cross-sectional study. PLoS One. 2016;11(6):e0157499. 10.1371/journal.pone.0157499 27310426 PMC4911164

[25] Rising KL, Powell RE, Cameron KA, et al. Development of the Uncertainty Communication Checklist: a patient-centered approach to patient discharge from the emergency department. Acad Med. 2020;95(7):1026–1034. 10.1097/ACM.0000000000003231 32101919 PMC7302334

[26] Using the Critical Incident Questionnaire (CIQ). Stephen D. Brookfield. Accessed June 28, 2021. http://www.stephenbrookfield.com/critical-incident-questionnaire

