# Supplementary material for: I Don't Have a Diagnosis for You: Preparing Medical Students to Communicate Diagnostic Uncertainty in the Emergency Department
Source: MedEdPORTAL. 2022 Feb 4;18:11218. doi: 10.15766/mep_2374-8265.11218 (PMC8814030; doi:10.15766/mep_2374-8265.11218)
Supplement: Supplementary file 1 — Uncertainty Communication Checklist.docxPrework Reflection Prompts.docxIntolerance of Uncertainty Scale.docxSelf-Compassion Scale Short Form.pdfUncertainty Articulate Module folderDebrief Facilitator Prompts.docxCommunicating Diagnostic Uncertainty Slides.pptxSimulation Student Role-Play Instructions.docxPostsession Survey.docx [file mep_2374-8265.11218-s001.zip › A. Uncertainty Communication Checklist.docx]

**Uncertainty Communication Checklist for Patient Discharge**

**From the Emergency Department**

**Introduction**

- 1. Explain to the patient that they are being discharged
- 2. Ask if there is anyone else whom the patient wishes to have included in the conversation in person and/or by phone

**Test results/ED summary**

- 3. Clearly state that either “life-threatening” or “dangerous” conditions have not been found
- 4. Discuss diagnoses that were considered (using both medical and lay terminology)
- 5. Communicate relevant results of tests to patients (normal or abnormal)
- 6. Ask patient if there are any questions about testing and/or results
- 7. Ask patient if they were expecting anything else to be done during their encounter—if yes, address reasons not done

**No/uncertain diagnosis**

- 8. Discuss possible alternate or working diagnoses
- 9. Clearly state that there is not a confirmed explanation (diagnosis) for what the patient has been experiencing
- 10. Validate the patient’s symptoms
- 11. Discuss that the ED role is to identify conditions that require immediate attention
- 12. Normalize leaving the ED with uncertainty

**Next steps/follow-up**

- 13. Suggest realistic expectations/trajectory for symptoms
- 14. Discuss next tests that are needed, if any
- 15. Discuss who to see next and in what time frame

**Home care**

- 16. Discuss a plan for managing symptoms at home
- 17. Discuss any medication changes
- 18. Ask patient if there are any questions and/or anticipated problems related to next steps (self-care and future medical care) after discharge

**Reasons to return**

- 19. Discuss what symptoms should prompt immediate return to the ED

**General communication skills**

- 20. Make eye contact
- 21. Ask patient if there are any other questions or concerns
- Abbreviation: ED, emergency department.

Adapted from Rising KL, Powell RE, Cameron KA, et al. Development of the uncertainty communication checklist: A patient-centered approach to patient discharge from the emergency department. *Acad Med*. 2020;95(7):1026-1034. <https://doi.org/10.1097/ACM.0000000000003231>
