# Supplementary material for: I Don't Have a Diagnosis for You: Preparing Medical Students to Communicate Diagnostic Uncertainty in the Emergency Department
Source: MedEdPORTAL. 2022 Feb 4;18:11218. doi: 10.15766/mep_2374-8265.11218 (PMC8814030; doi:10.15766/mep_2374-8265.11218)
Supplement: Supplementary file 1 — Uncertainty Communication Checklist.docxPrework Reflection Prompts.docxIntolerance of Uncertainty Scale.docxSelf-Compassion Scale Short Form.pdfUncertainty Articulate Module folderDebrief Facilitator Prompts.docxCommunicating Diagnostic Uncertainty Slides.pptxSimulation Student Role-Play Instructions.docxPostsession Survey.docx [file mep_2374-8265.11218-s001.zip › B. Prework Reflection Prompts.docx]

**Pre-work Reflection Prompts**

Students often encounter uncertainty during the transition into the clinical environment. One’s comfort or tolerance for uncertainty can have profound effects on clinicians working in the clinical environment. During this session, we want to spend time discussing the impact uncertainty has had on you during your clerkship experiences. Please take a moment to reflect on the uncertainty you may have encountered over the course of the last year. Below you will find 3 prompts to help guide your reflection.

**Describe a time you dealt with uncertainty in a clinical situation.**

**Why was this important? How did this make you feel?**

**What did you take away? How has this impacted you?**
