# Supplementary material for: I Don't Have a Diagnosis for You: Preparing Medical Students to Communicate Diagnostic Uncertainty in the Emergency Department
Source: MedEdPORTAL. 2022 Feb 4;18:11218. doi: 10.15766/mep_2374-8265.11218 (PMC8814030; doi:10.15766/mep_2374-8265.11218)
Supplement: Supplementary file 1 — Uncertainty Communication Checklist.docxPrework Reflection Prompts.docxIntolerance of Uncertainty Scale.docxSelf-Compassion Scale Short Form.pdfUncertainty Articulate Module folderDebrief Facilitator Prompts.docxCommunicating Diagnostic Uncertainty Slides.pptxSimulation Student Role-Play Instructions.docxPostsession Survey.docx [file mep_2374-8265.11218-s001.zip › D. Self-Compassion Scale Short Form.pdf]

Self-Compassion Scale Short Form (SCS-SF)

HOW I TYPICALLY ACT TOWARDS MYSELF IN DIFFICULT TIMES

Please read each statement carefully before answering. Indicate how often you behave in the stated manner, using the following scale:

Almost never				Almost always
**1**	**2**	**3**	**4**	**5**

1. When I fail at something important to me I become consumed by feelings of inadequacy.

2. I try to be understanding and patient towards those aspects of my personality I don't like.

3. When something painful happens I try to take a balanced view of the situation.

4. When I'm feeling down, I tend to feel like most other people are probably happier than I am.

5. I try to see my failings as part of the human condition.

6. When I'm going through a very hard time, I give myself the caring and tenderness I need.

7. When something upsets me I try to keep my emotions in balance.

8. When I fail at something that's important to me, I tend to feel alone in my failure

9. When I'm feeling down I tend to obsess and fixate on everything that's wrong.

10. When I feel inadequate in some way, I try to remind myself that feelings of inadequacy are shared by most people.

11. I'm disapproving and judgmental about my own flaws and inadequacies.

12. I'm intolerant and impatient towards those aspects of my personality I don't like.

Used with permission from Dr. Kristen Neff.

Reference:

Raes, F., Pommier, E., Neff, K. D., & Van Gucht, D. (2011). Construction and factorial validation of a short form of the Self-Compassion Scale. *Clinical Psychology & Psychotherapy*. 18, 250-255

SCORING KEY
Self-Kindness Items: 2, 6
Self-Judgment Items (Reverse Scored): 11, 12
Common Humanity Items: 5, 10
Isolation Items (Reverse Scored): 4, 8
Mindfulness Items: 3, 7
Over-identification Items (Reverse Scored): 1, 9

To reverse score items (1=5, 2=4, 3=3, 4=2, 5=1).

To compute a total self-compassion score, first reverse score the negative subscale items - self-judgment, isolation, and over-identification. Then take the mean of each subscale, and compute a total mean (the average of the six subscale means).

Note that these scoring procedures are slightly different than that used in the original scale article (Raes et al., 2011), in which items were totaled rather than averaged. However, it is easier to interpret the scores of the total mean is used and most researchers currently report total SCS-SF scores on a five-point scale.

NORMS AND SCORE SIGNIFICANCE

There are no clinical norms or scores which indicate that an individual is high or low in self-compassion. Rather, scores are mainly used in a comparative manner to examine outcomes for people scoring higher or lower in self-compassion. As an ad hoc rubric, however, you can consider scores 1.0-2.49 to be low, between 2.5-3.5 to be moderate, and 3.51-5.0 to be high. When trying to determine whether self-compassion levels are high or low relevant to a particular sample, some researchers use a median split.

SCALE DEVELOPMENT, RELIABILITY AND VALIDITY

Raes et al. (2011) developed a short form of the SCS containing 12 of the original 26 SCS items. A Dutch sample (n=271) was used to construct the SCS-SF. To create the scale, two items from each of the six self-compassion subscales were selected that demonstrated high correlations with the long SCS total score and high correlations with their intended SCS subscale. A second Dutch sample (n=185) was used to validate the factor structure of the SCS-SF. Confirmatory factor analyses supported the same correlated six-factor structure as found in the original study of the long form (Neff, 2003a), as well as a single higher-order factor of self-compassion. The SCS-SF was then validated in a third, English sample (n=415). The six-factor structure and a single higher-order factor was replicated. The SCS-SF demonstrated adequate internal consistency (Cronbach's alpha $\geq .86$ in all samples) and a near-perfect correlation with the long form SCS ($r \geq .97$ all samples). The SCS-SF is a reliable alternative to the long form SCS, especially when looking at overall self-compassion scores. Because each subscale only contains two items, however, reliability of the subscales is lower (*r*'s ranging from .54 - .75). Because subscale scores are unreliable in the SCS short form, we recommend using the original SCS rather than the short form when examining subscale scores.

ANALYTIC APPROACH FOR VALIDATION AND TRANSLATION

In order to validate the factor structure of the scale (including for translations) we strongly recommend the use of bifactor ESEM, as this is the most appropriate method to assess the operation of self-compassion components as a system. Information on this analytic method can be found in (Neff et al., 2019). Moreover, appropriate syntax for how to conduct these analyses for the SCS using Mplus can be found in the online supplement to that article and also here.

Many translations of the full SCS already exist can be found here. The 12 SCS-SF items are taken from the full 26 item SCS. If you would like to use a translation of the SCS-SF, you might try just taking the 12 short form items from a previously translated version of the long form.

Short SCS	Long SCS
1	6
2	26
3	14
4	13
5	15
6	12
7	9
8	25
9	2
10	10
11	1
12	11

Used with permission from Dr. Kristen Neff.

Reference:
Neff, K. D., Tóth-Király, I., Yarnell, L., Arimitsu, K., Castilho, P., Ghorbani, N.,… Mantios, M. (2019). Examining the Factor Structure of the Self-Compassion Scale using exploratory SEM bifactor analysis in 20 diverse samples: Support for use of a total score and six subscale scores. Psychological Assessment, 31 (1), 27-45.
