# Supplementary material for: I Don't Have a Diagnosis for You: Preparing Medical Students to Communicate Diagnostic Uncertainty in the Emergency Department
Source: MedEdPORTAL. 2022 Feb 4;18:11218. doi: 10.15766/mep_2374-8265.11218 (PMC8814030; doi:10.15766/mep_2374-8265.11218)
Supplement: Supplementary file 1 — Uncertainty Communication Checklist.docxPrework Reflection Prompts.docxIntolerance of Uncertainty Scale.docxSelf-Compassion Scale Short Form.pdfUncertainty Articulate Module folderDebrief Facilitator Prompts.docxCommunicating Diagnostic Uncertainty Slides.pptxSimulation Student Role-Play Instructions.docxPostsession Survey.docx [file mep_2374-8265.11218-s001.zip › E. Uncertainty Articulate Module/assets/asset citations.docx]

**Citations for resources used in Appendix E, Uncertainty Articulate Module:**

| **Asset Name** | **Thumbnail** | **Citation** |
| --- | --- | --- |
| -lzrDbCcLofpDbAW_ul0W2h1SpUEmX5Yc.png | *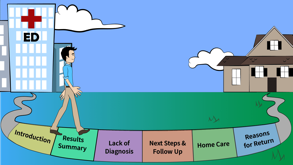* | Author created |
| -Mowi3aii6bpzhL7_quote_background.jpg | 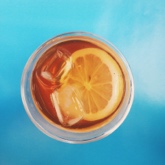 | Image by Julia D’Alkmin, retrieved from <https://unsplash.com/photos/WKCjwrtjhVg> on 7/1/21. Free to use without attribution under Unsplash license: lhttps://unsplash.com/license |
| 0AtEVmwk_7OCc9wR_transcoded-57RicWYLfoOx9S_V-1-intro-bad | *Audio file* | Author created |
| 0HY5cIqxbhVVDfLD_VK8sFjhMOMDK7q3n.jpg | *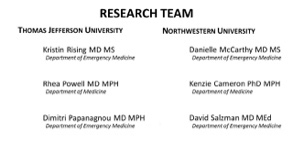* | Author created |
| 6ZjksOE-wadkIBhJ_pAoAAhKluVB7q1g0.png | *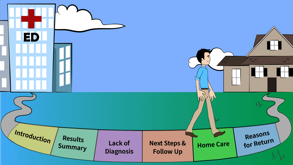* | Author created |
| 93LJW2IFLoxzGePI_transcoded-En-8WZ8vFTyBgrAU-4-next-steps-bad.mp3 | *Audio file* | Author created |
| CH80MN5fIiaycTgT_mountains.jpg | 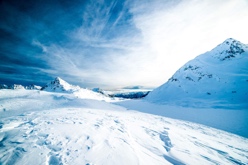 | Image by Alberto Restifo, retrieved from <https://unsplash.com/photos/cFplR9ZGnAk> on 7/1/21. Free to use without attribution under Unsplash license: lhttps://unsplash.com/license |
| cRXbGLAl1a51UZMp_transcoded-oyPsdQ2fDghw5brH-2-results-good.mp3 | *Audio file* | Author created |
| F7_7tuFSVOeq9huf_transcoded-dfJx4rvjXCXDKt9P-5-home-care-good.mp3 | *Audio file* | Author created |
| fwu4mimwpYNgi8Yj_transcoded-J52U34lGVT4CdNha-5-home-care-bad.mp3 | *Audio file* | Author created |
| GpVYPovXn6pLD3nz_transcoded-Ck610hoX8wtijToo-6-reasons-return-bad.mp3 | *Audio file* | Author created |
| gUK3LgukhK5PnaUa_transcoded-u2x-hWlLNVZMwoJ9-Video 1_Challenges for Doctors Module-00001.png | *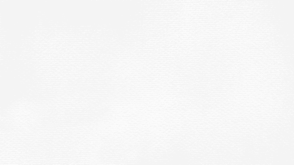*  *(image)* | Author created  (created with premium video scribe software license (Sparkol), which allows users to design, develop and publish original content without attribution) |
| i6ZdcgOnDOYlkenw_transcoded-u2x-hWlLNVZMwoJ9-Video 1_Challenges for Doctors Module.mp4 | *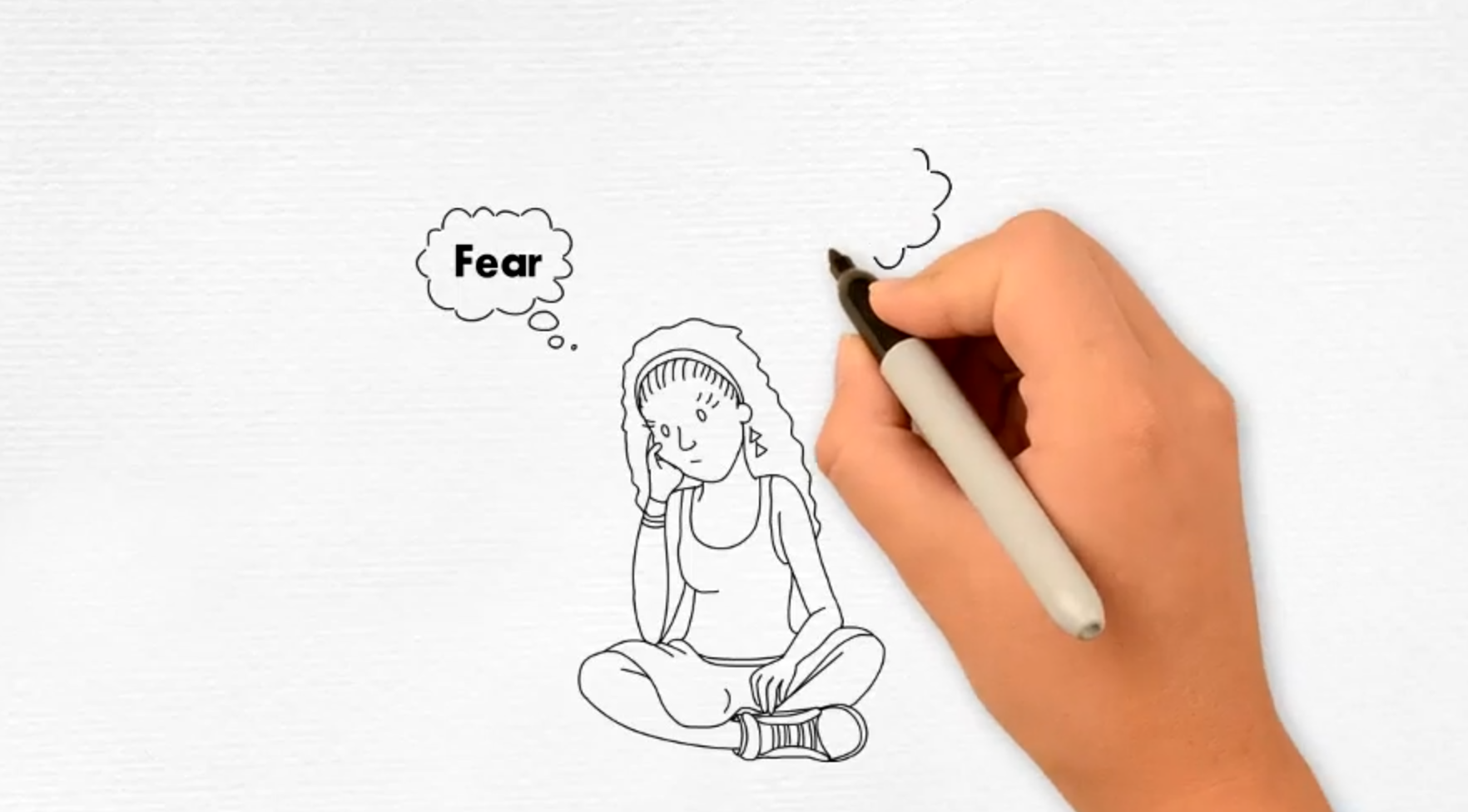*  *(video file)* | Author created  (created with premium video scribe software license (Sparkol), which allows users to design, develop and publish original content without attribution) |
| IDjtvIPDoaZsTI59_2Jiv7S8oxMXGJutm-stock-image.jpg | 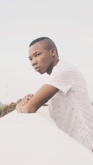 | Image by Axel Mencia, retrieved from <https://unsplash.com/photos/TcoY45y4IxU> on 7/1/21. Free to use without attribution under Unsplash license: lhttps://unsplash.com/license |
| jHiV0C6yNeJVkaA5_transcoded-GCI6om_nPjUN46gJ-3-uncertain-dx-bad.mp3 | *Audio file* | Author created |
| LOmk4_a5MEBAKLnb_small_1579644331.png | *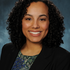* | Author created |
| m29UIoNp2Jsuw4NT_transcoded-4FcG8WDNwk-mpS8y-Video 2_Patient Concerns_Putting it all Together Module.mp4 | *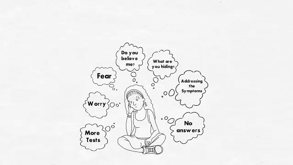*  *(video file)* | Author created  (created with premium video scribe software license (Sparkol), which allows users to design, develop and publish original content without attribution) |
| M8fA6TlZMdKgX0H6_transcoded-Upl_caRdIqMQffjM-2-results-bad.mp3 | *Audio file* | Author created |
| nrCzV5NdSWe-1k7a_OBeqI1VxGhY-zevR.png | 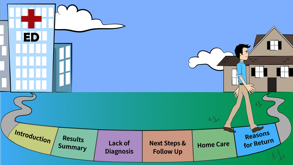 | Author created |
| nVurpl4K9BwvAI_i_transcoded-n3gr87lKU4WH5BlJ-4-next-steps-good.mp3 | *Audio file* | Author created |
| pB4uSDF8xDxyylgQ_jTVSwAmBghLZmkmK.png | 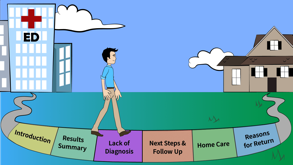 | Author created |
| POqJV_YTERweItbm_transcoded-SIYfK6MQmYXDs12y-3-uncertain-dx-good.mp3 | *Audio file* | Author created |
| slRFem0EqQbuxDdP_qMwaHn0ruZgBED0K-stock-image.jpg | *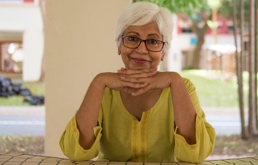* | Image by, retrieved from on 7/1/21. Free to use without attribution under Unsplash license: lhttps://unsplash.com/license |
| T3qSVhOMq1oyGKVa_wWPtEhntRYzamMIT.png | 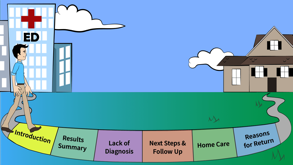 | Author created |
| ThUtFwP3Z9FhREwA_transcoded-s1hfyexWhW2XmySN-1-intro-good.mp3 | *Audio file* | Author created |
| tmzRr97hixn6W3UC_OyTqSJWe2kSA-R5y-stock-image.jpg | 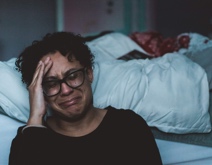 | Image by Claudia Wolff, retrieved from <https://unsplash.com/photos/owBcefxgrIE> on 7/1/21. Free to use without attribution under Unsplash license: lhttps://unsplash.com/license |
| tNRzxOq2GusfioVf_transcoded-4FcG8WDNwk-mpS8y-Video 2_Patient Concerns_Putting it all Together Module-00001.png | *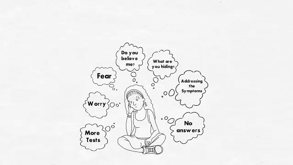*  *(image)* | Author created  (created with premium video scribe software license (Sparkol), which allows users to design, develop and publish original content without attribution) |
| UO1MGH-xTr9OUckJ_transcoded-KmDFW_lFPbTqE43B-6-reasons-return-good.mp3 | *Audio file* | Author created |
| VjKNjClCmbEiYfP1_small.png | 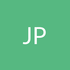 | Author created |
| vRBqCwKWNiEb7EMY_small.png | 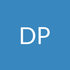 | Author created |
| xYfAaakrRi-GHnoY_Z39pTc1JXu1_tBNa-stock-image.jpg | 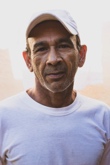 | Image by Ramille Soares, retrieved from <https://unsplash.com/photos/cyOKLSgkgCE>  on 7/1/21. Free to use without attribution under Unsplash license: lhttps://unsplash.com/license |
| z5-Im-gRQYWGPyhV_mv7W3-0ZNiBQjRin-stock-image.jpg | *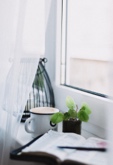* | Image by Daiga Ellaby, retrieved from <https://unsplash.com/photos/05XkxdF5dhw> on 7/1/21. Free to use without attribution under Unsplash license: lhttps://unsplash.com/license |
| ZjjHPsyDI796D76D_vk8PmAI6NTwukVc0.png | 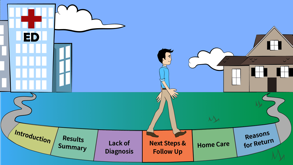 | Author created |
