# Supplementary material for: I Don't Have a Diagnosis for You: Preparing Medical Students to Communicate Diagnostic Uncertainty in the Emergency Department
Source: MedEdPORTAL. 2022 Feb 4;18:11218. doi: 10.15766/mep_2374-8265.11218 (PMC8814030; doi:10.15766/mep_2374-8265.11218)
Supplement: Supplementary file 1 — Uncertainty Communication Checklist.docxPrework Reflection Prompts.docxIntolerance of Uncertainty Scale.docxSelf-Compassion Scale Short Form.pdfUncertainty Articulate Module folderDebrief Facilitator Prompts.docxCommunicating Diagnostic Uncertainty Slides.pptxSimulation Student Role-Play Instructions.docxPostsession Survey.docx [file mep_2374-8265.11218-s001.zip › E. Uncertainty Articulate Module/index.html]

Communicating Diagnostic Uncertainty
