# Supplementary material for: I Don't Have a Diagnosis for You: Preparing Medical Students to Communicate Diagnostic Uncertainty in the Emergency Department
Source: MedEdPORTAL. 2022 Feb 4;18:11218. doi: 10.15766/mep_2374-8265.11218 (PMC8814030; doi:10.15766/mep_2374-8265.11218)
Supplement: Supplementary file 1 — Uncertainty Communication Checklist.docxPrework Reflection Prompts.docxIntolerance of Uncertainty Scale.docxSelf-Compassion Scale Short Form.pdfUncertainty Articulate Module folderDebrief Facilitator Prompts.docxCommunicating Diagnostic Uncertainty Slides.pptxSimulation Student Role-Play Instructions.docxPostsession Survey.docx [file mep_2374-8265.11218-s001.zip › F. Debrief Facilitator Prompts.docx]

**Facilitator Prompts**

*Following the role-play, students will enter breakout rooms of between eight and twelve students for debriefing. One faculty member should enter each breakout room to facilitate the debrief session. Facilitators should encourage students to discuss their experience of the role-play, and to hear from students who played the role of the patient, provider, and observer. Use the below prompts to help scaffold the discussion and elicit reflective responses.*

**At what moment during the role play did you feel most engaged with what was happening?**

**At what moment during the role play did you feel most distanced from what was happening?**

**What action(s) during the role play did you find most affirming or helpful?**

**What action(s) during the role play did you find most puzzling or confusing?**

**What about the role play surprised you the most? (This could be about your own reactions to what went on, something that someone did, or anything else that occurred).**

**How did the role play change or reaffirm your views on communicating diagnostic uncertainty?**
