# Supplementary material for: I Don't Have a Diagnosis for You: Preparing Medical Students to Communicate Diagnostic Uncertainty in the Emergency Department
Source: MedEdPORTAL. 2022 Feb 4;18:11218. doi: 10.15766/mep_2374-8265.11218 (PMC8814030; doi:10.15766/mep_2374-8265.11218)
Supplement: Supplementary file 1 — Uncertainty Communication Checklist.docxPrework Reflection Prompts.docxIntolerance of Uncertainty Scale.docxSelf-Compassion Scale Short Form.pdfUncertainty Articulate Module folderDebrief Facilitator Prompts.docxCommunicating Diagnostic Uncertainty Slides.pptxSimulation Student Role-Play Instructions.docxPostsession Survey.docx [file mep_2374-8265.11218-s001.zip › G. Communicating Diagnostic Uncertainty Slides.pptx]

## Slide 1
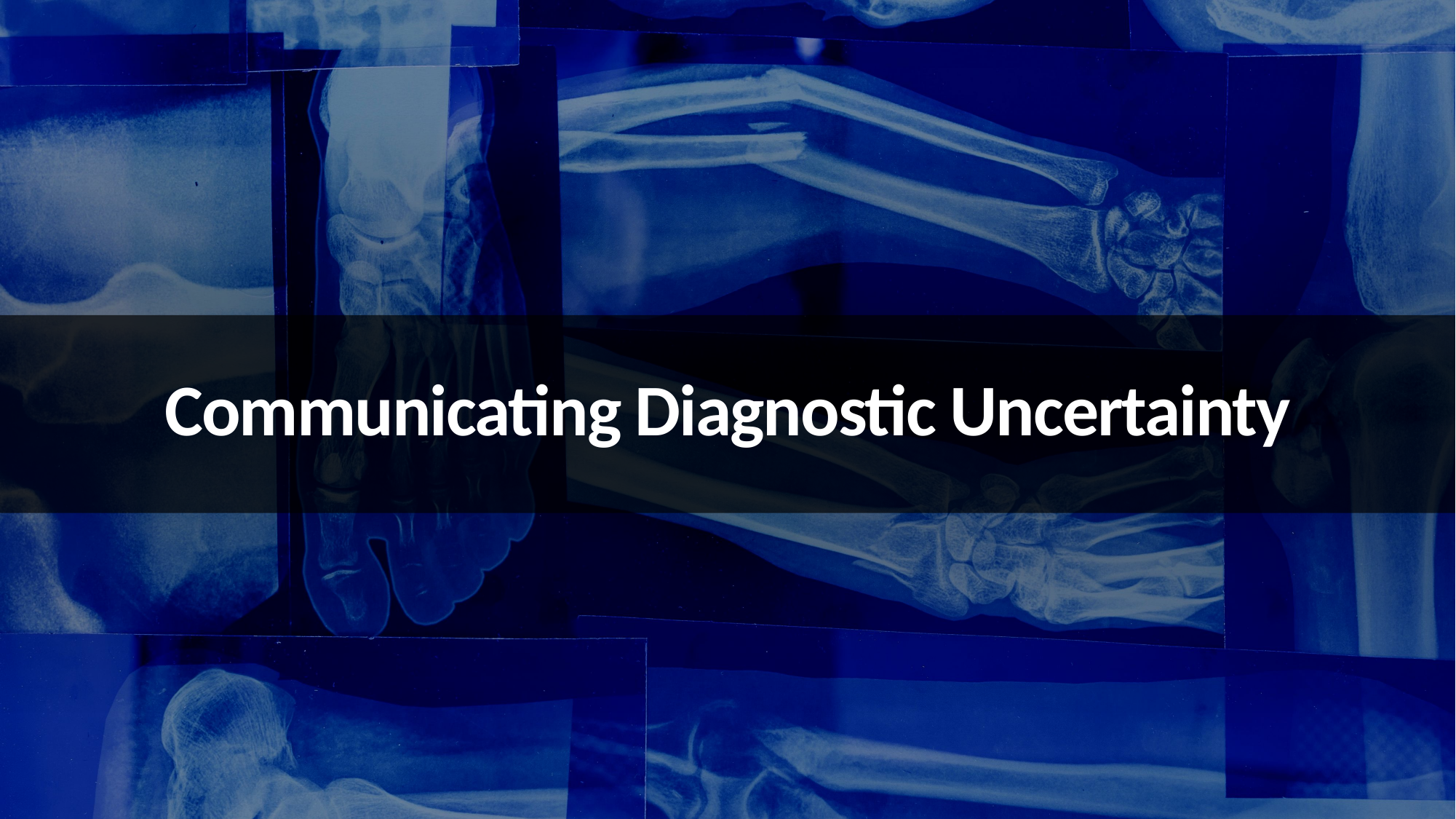

# Communicating Diagnostic Uncertainty

## Slide 2
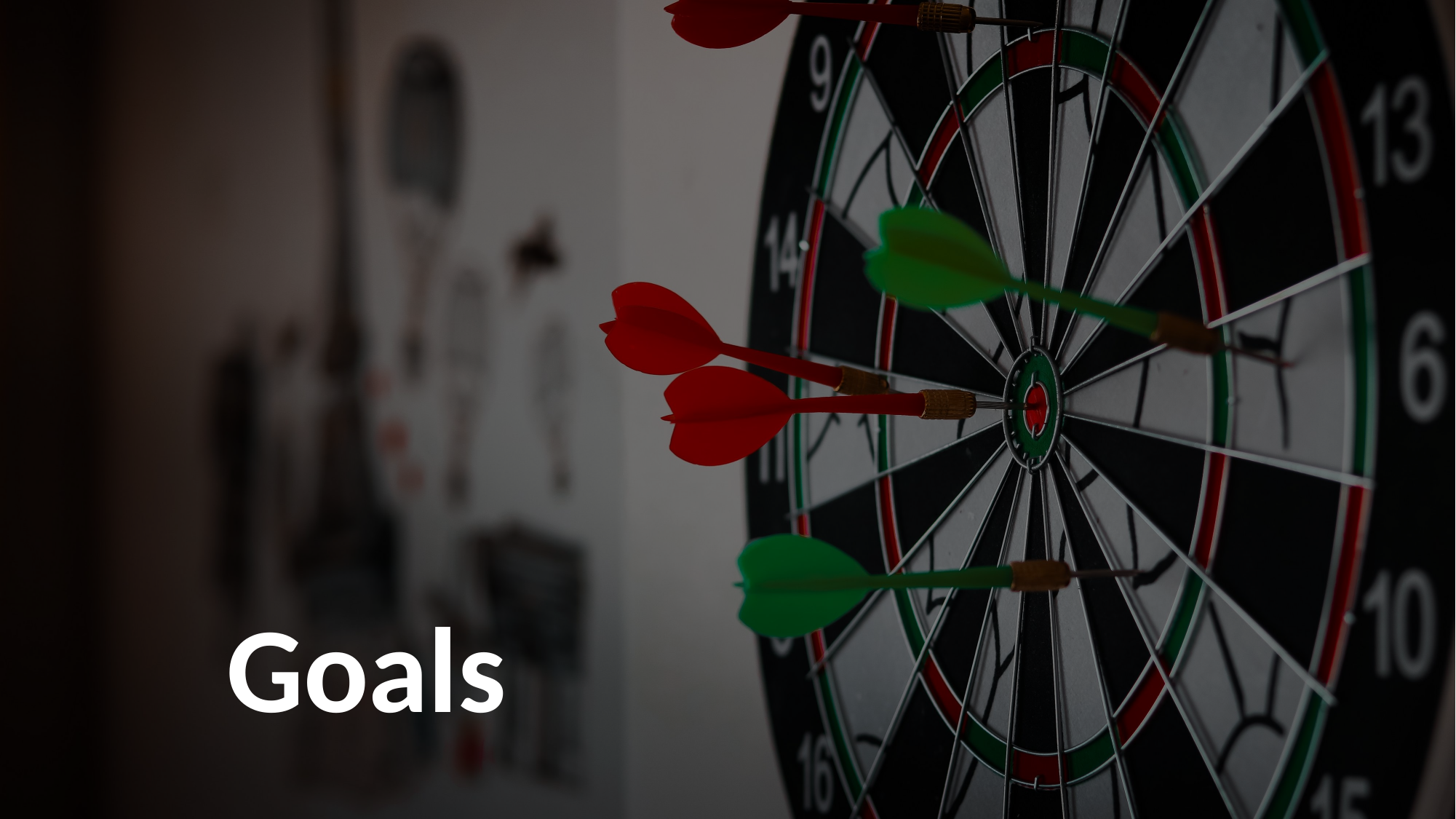

# Goals

## Slide 3
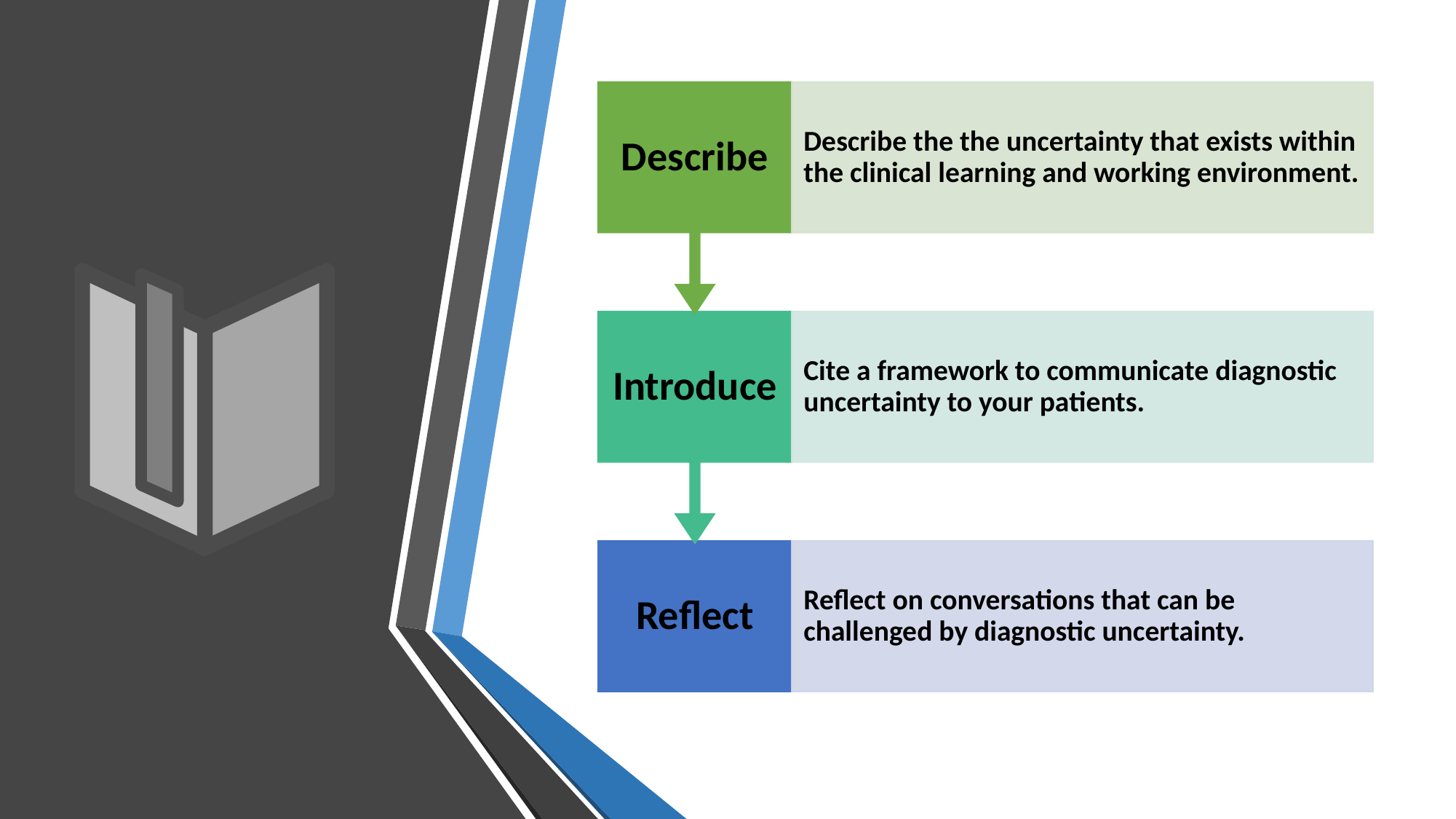

## Slide 4
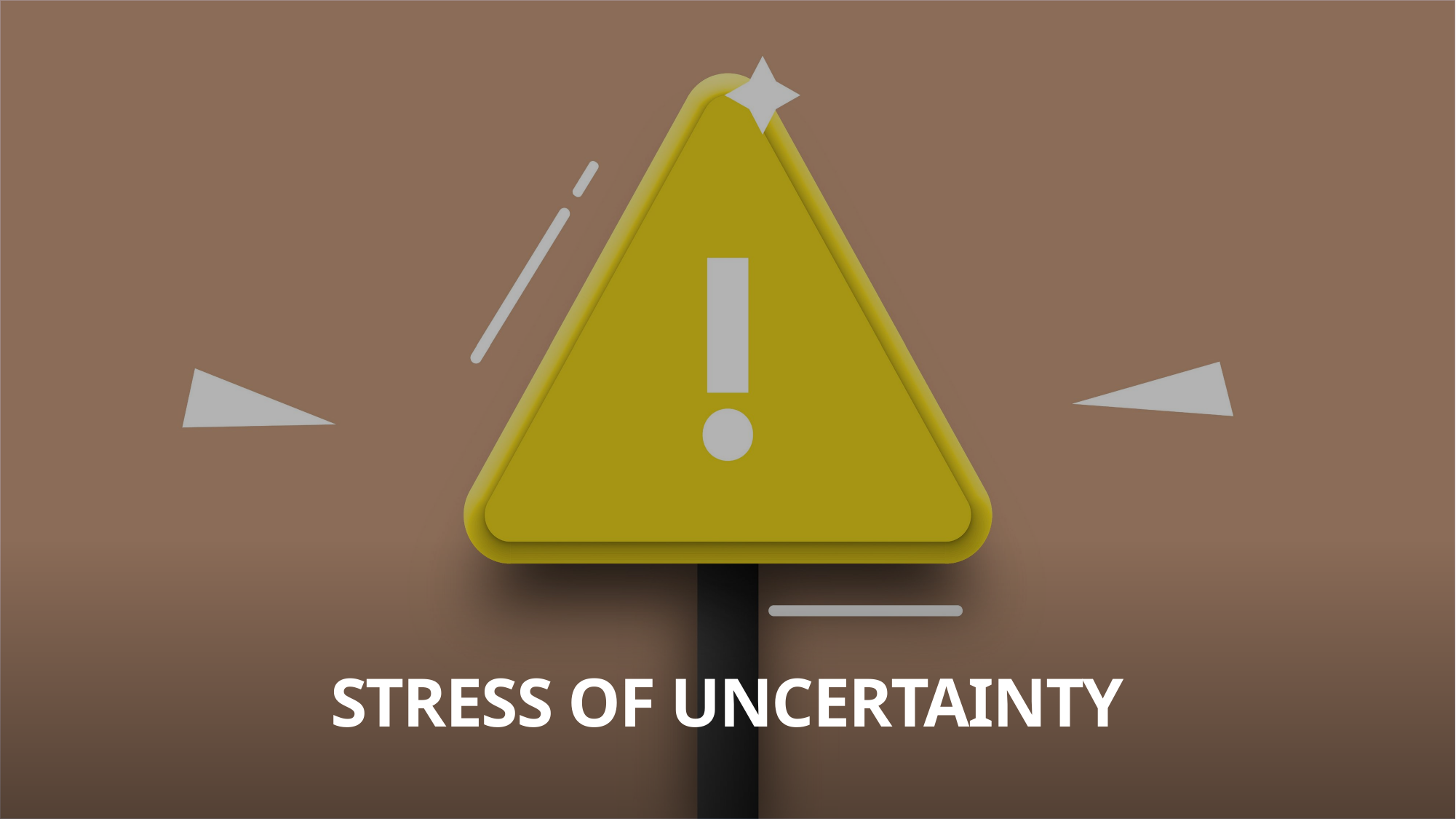

# Stress of Uncertainty

## Slide 5
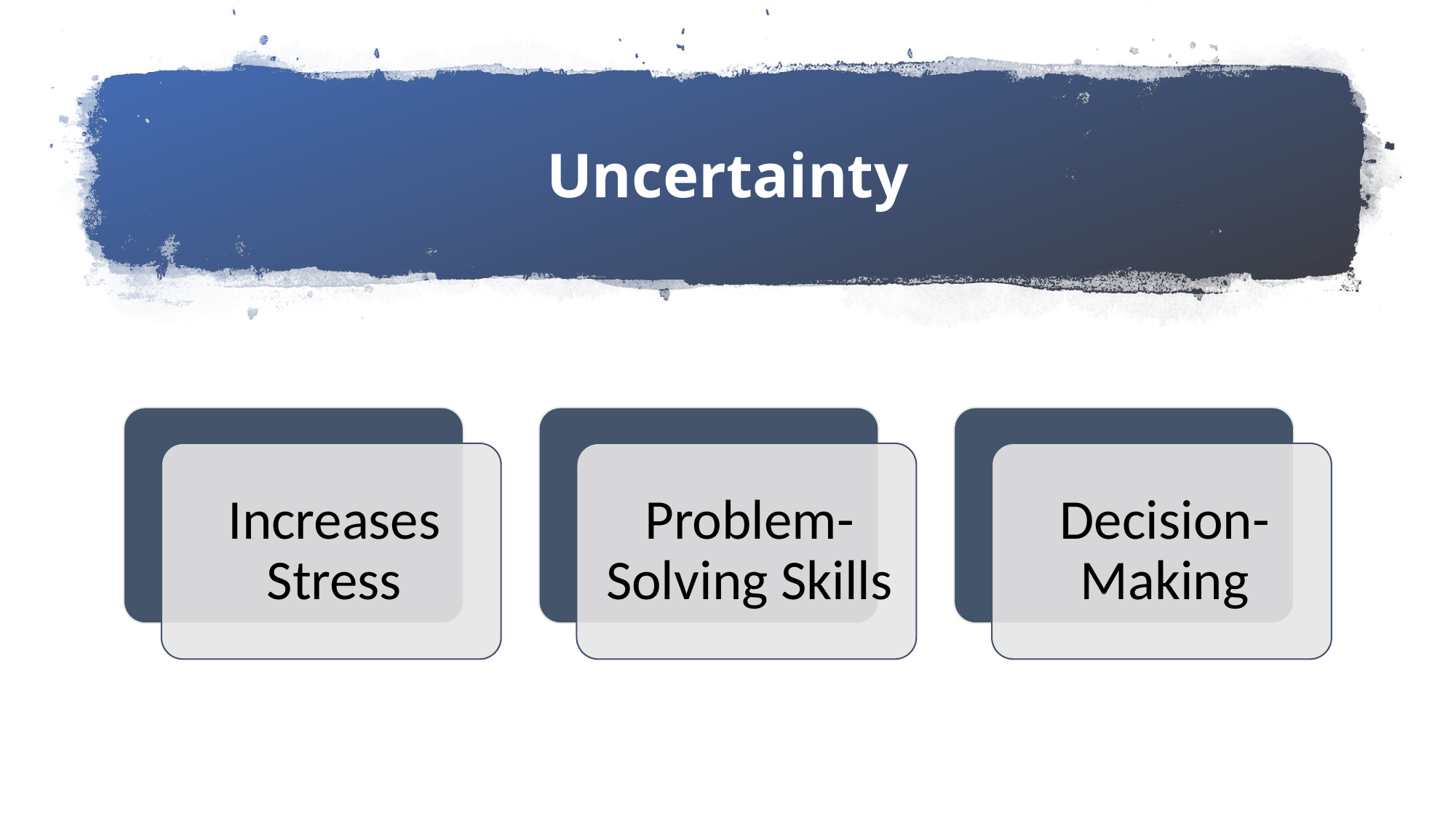

# Uncertainty

## Slide 6
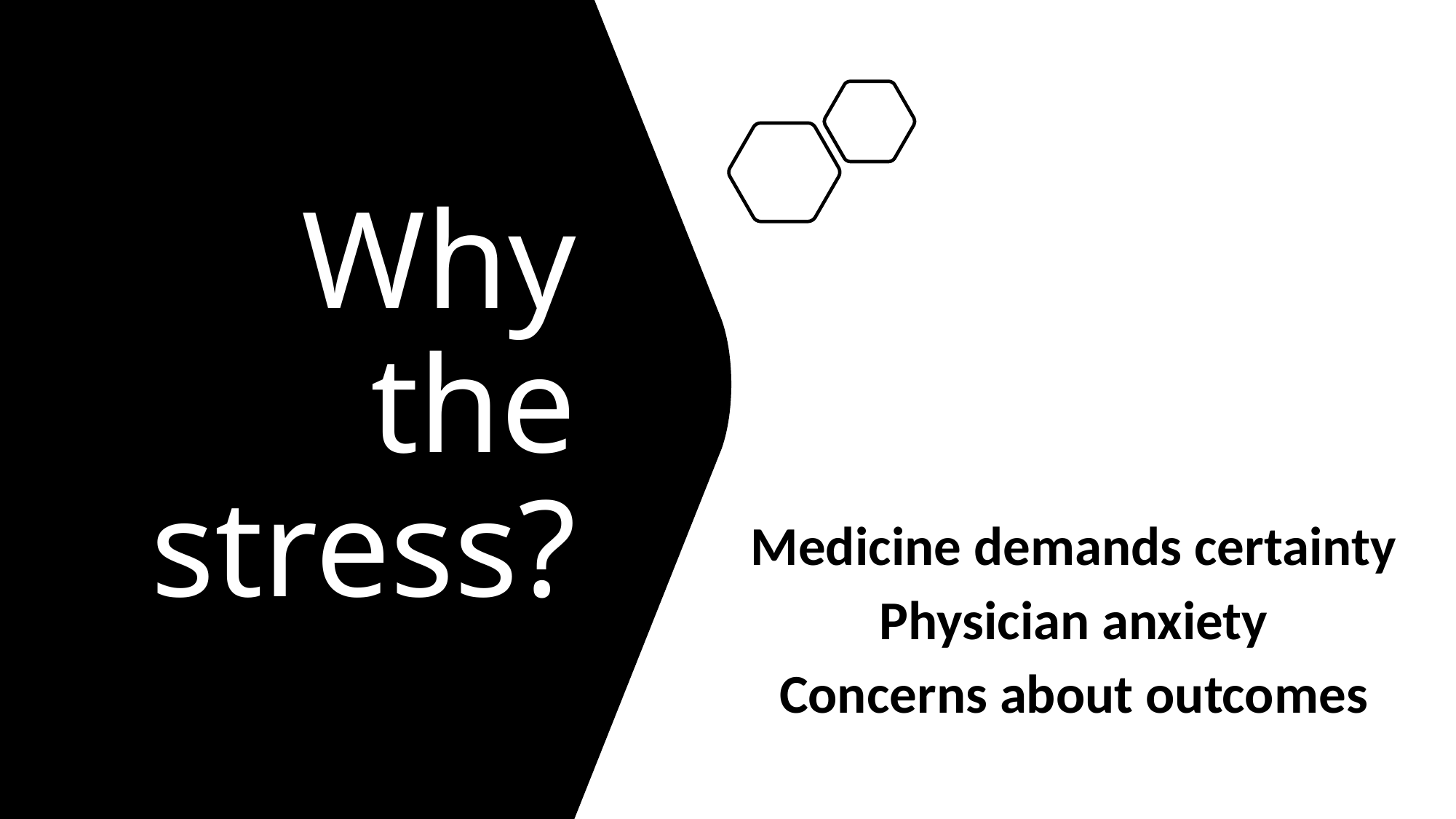

# Why the stress?
Medicine demands certainty
Physician anxiety
Concerns about outcomes

## Slide 7
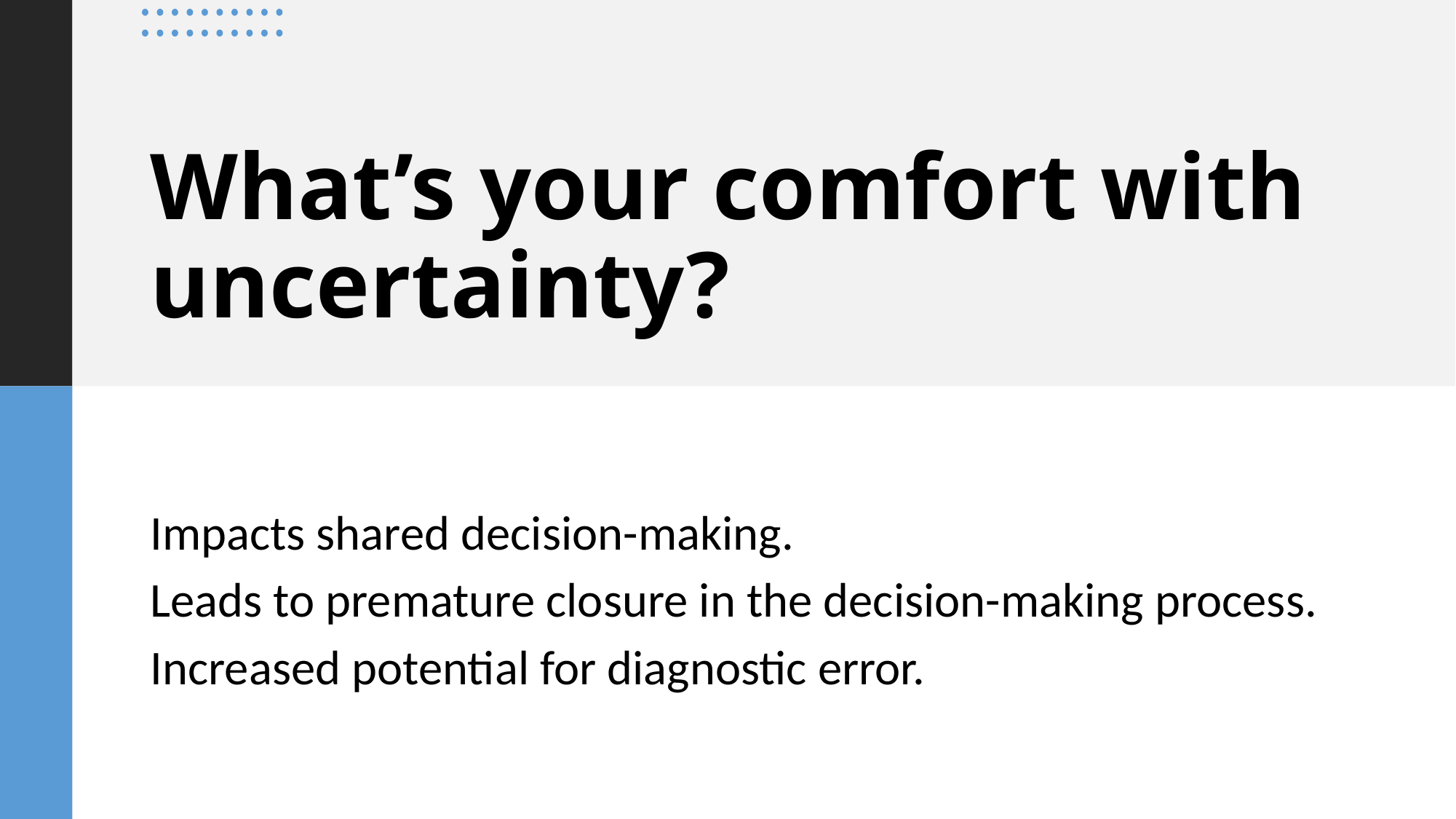

# What’s your comfort with uncertainty?
Impacts shared decision-making.
Leads to premature closure in the decision-making process.
Increased potential for diagnostic error.

## Slide 8
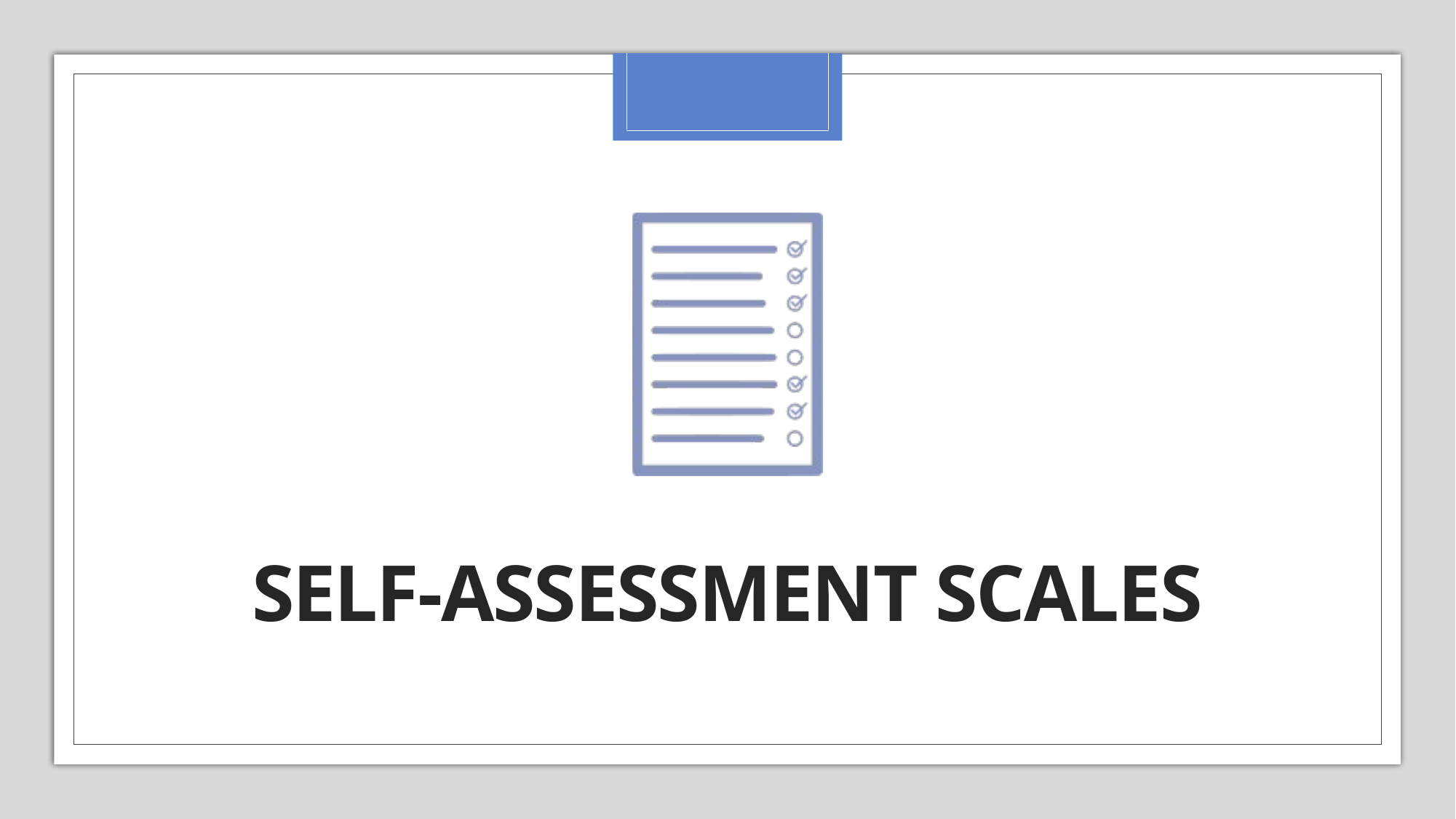

# Self-Assessment Scales

## Slide 9
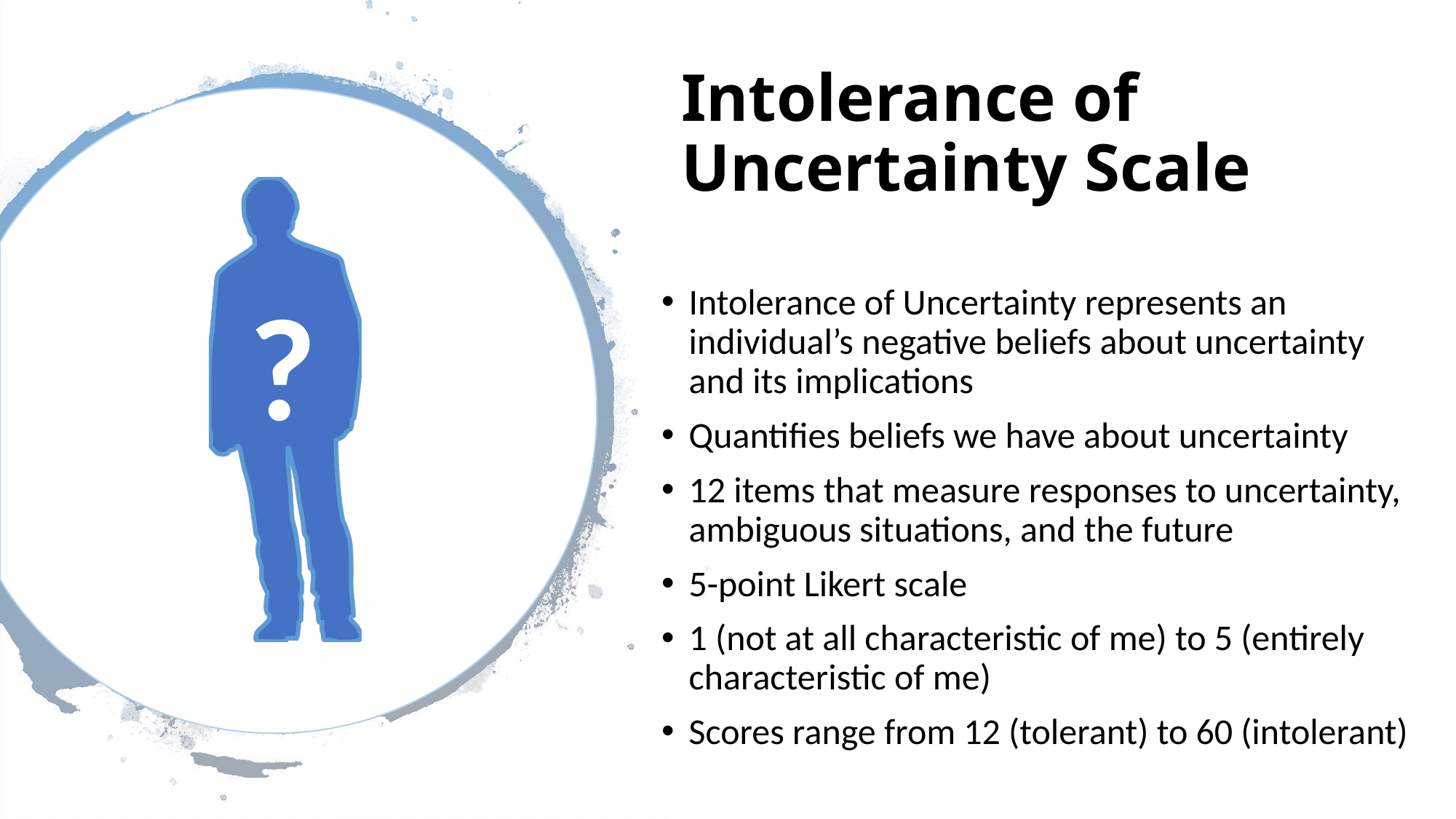

# Intolerance of Uncertainty Scale
?
Intolerance of Uncertainty represents an individual’s negative beliefs about uncertainty and its implications
Quantifies beliefs we have about uncertainty
12 items that measure responses to uncertainty, ambiguous situations, and the future
5-point Likert scale
1 (not at all characteristic of me) to 5 (entirely characteristic of me)
Scores range from 12 (tolerant) to 60 (intolerant)

## Slide 10
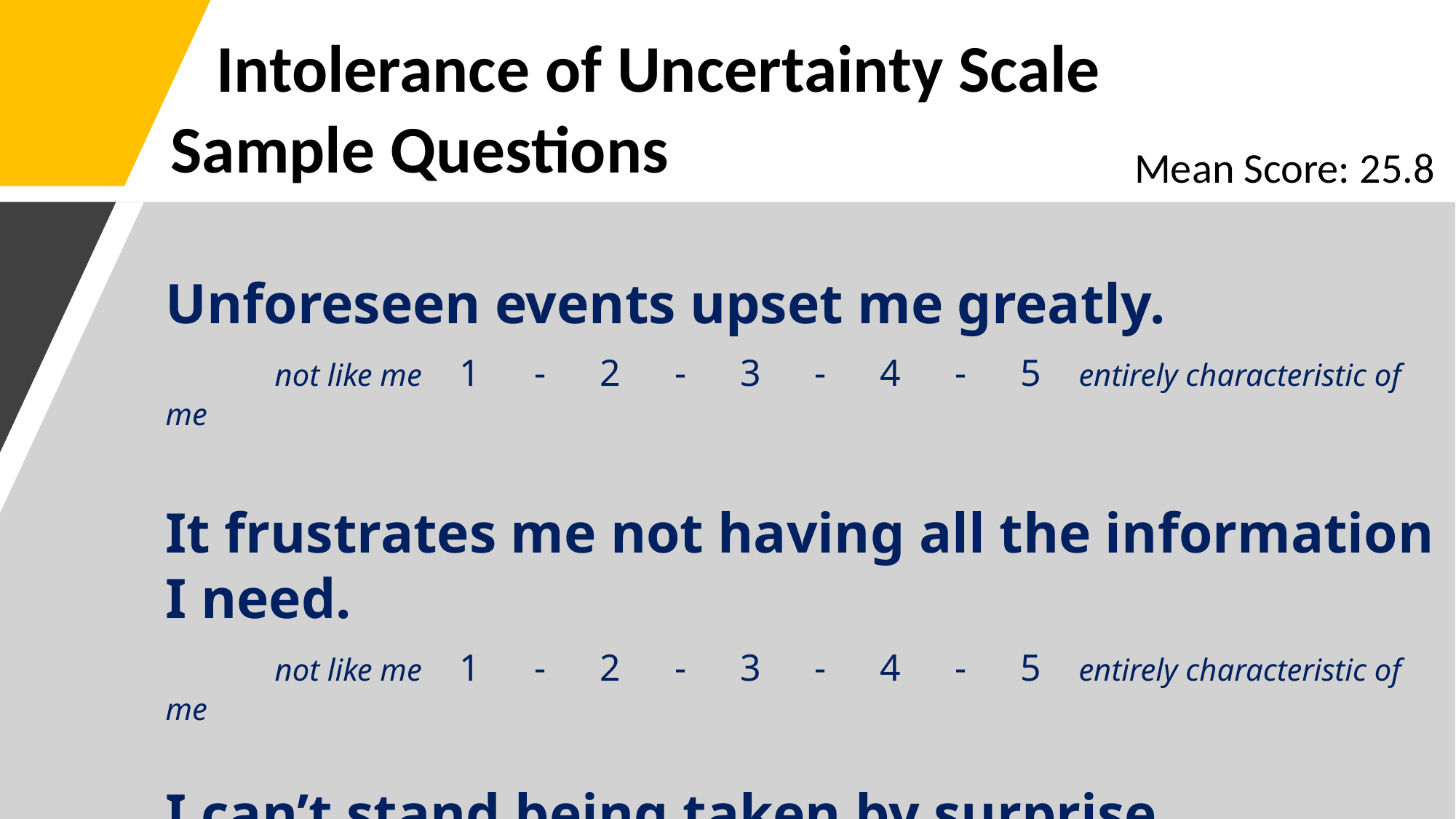

Intolerance of Uncertainty Scale
Sample Questions
Mean Score: 25.8
Unforeseen events upset me greatly.
 	not like me 1　-　2　-　3　-　4　-　5 entirely characteristic of me
It frustrates me not having all the information I need.
	not like me 1　-　2　-　3　-　4　-　5 entirely characteristic of me
I can’t stand being taken by surprise.
 	not like me 1　-　2　-　3　-　4　-　5 entirely characteristic of me

## Slide 11
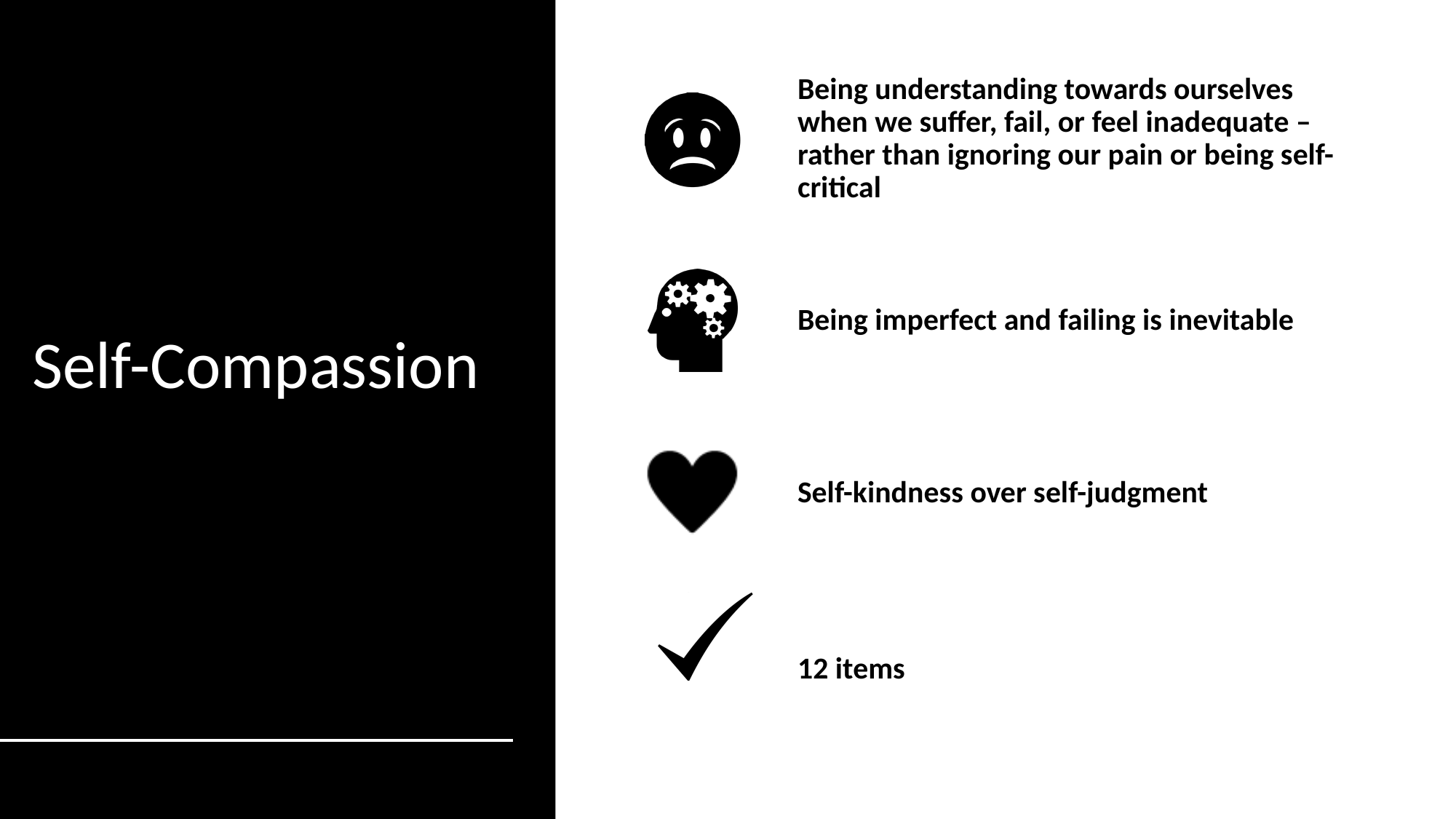

Being understanding towards ourselves when we suffer, fail, or feel inadequate – rather than ignoring our pain or being self-critical
# Self-Compassion
Being imperfect and failing is inevitable
Self-kindness over self-judgment
12 items

## Slide 12
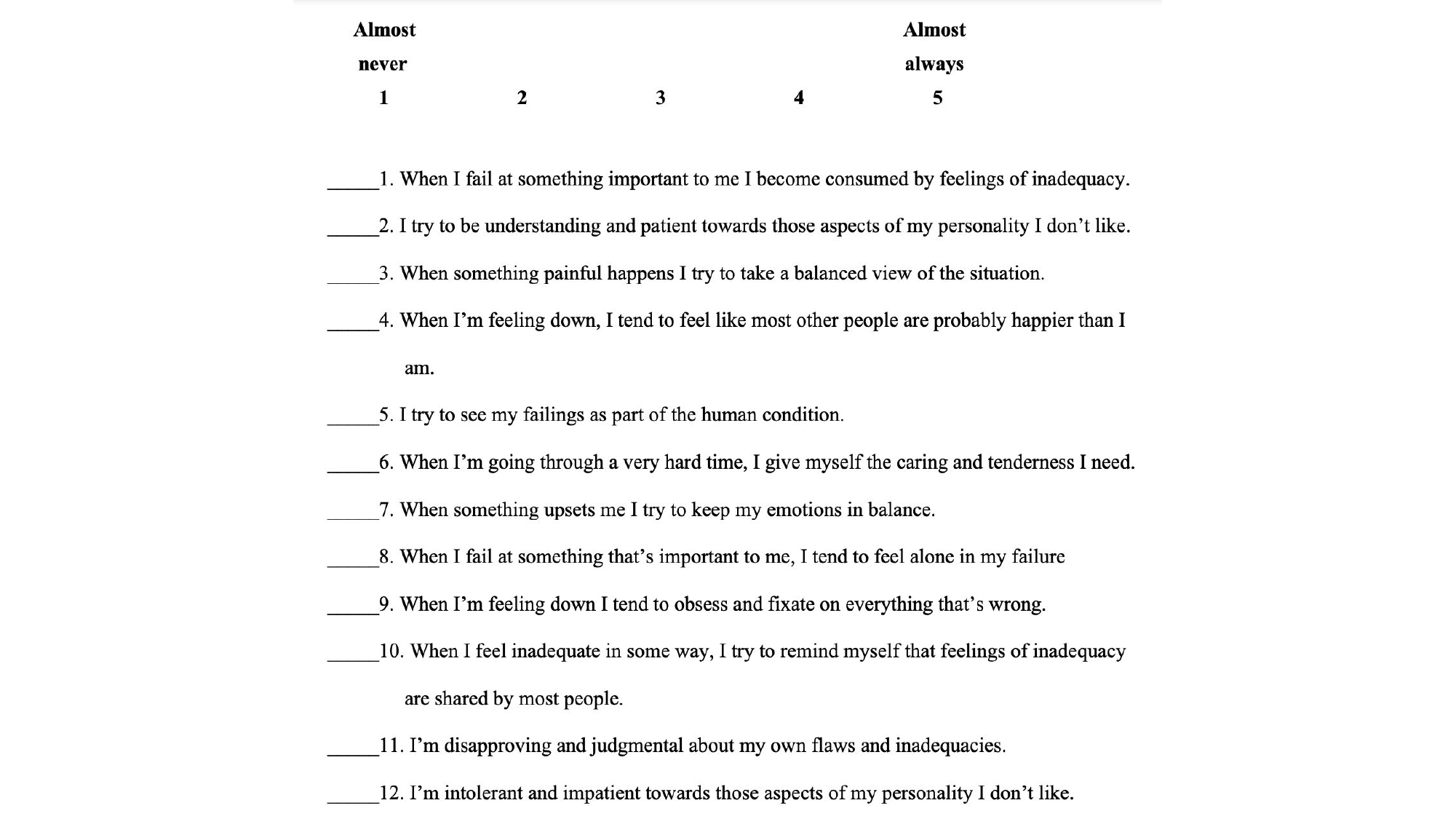

## Slide 13
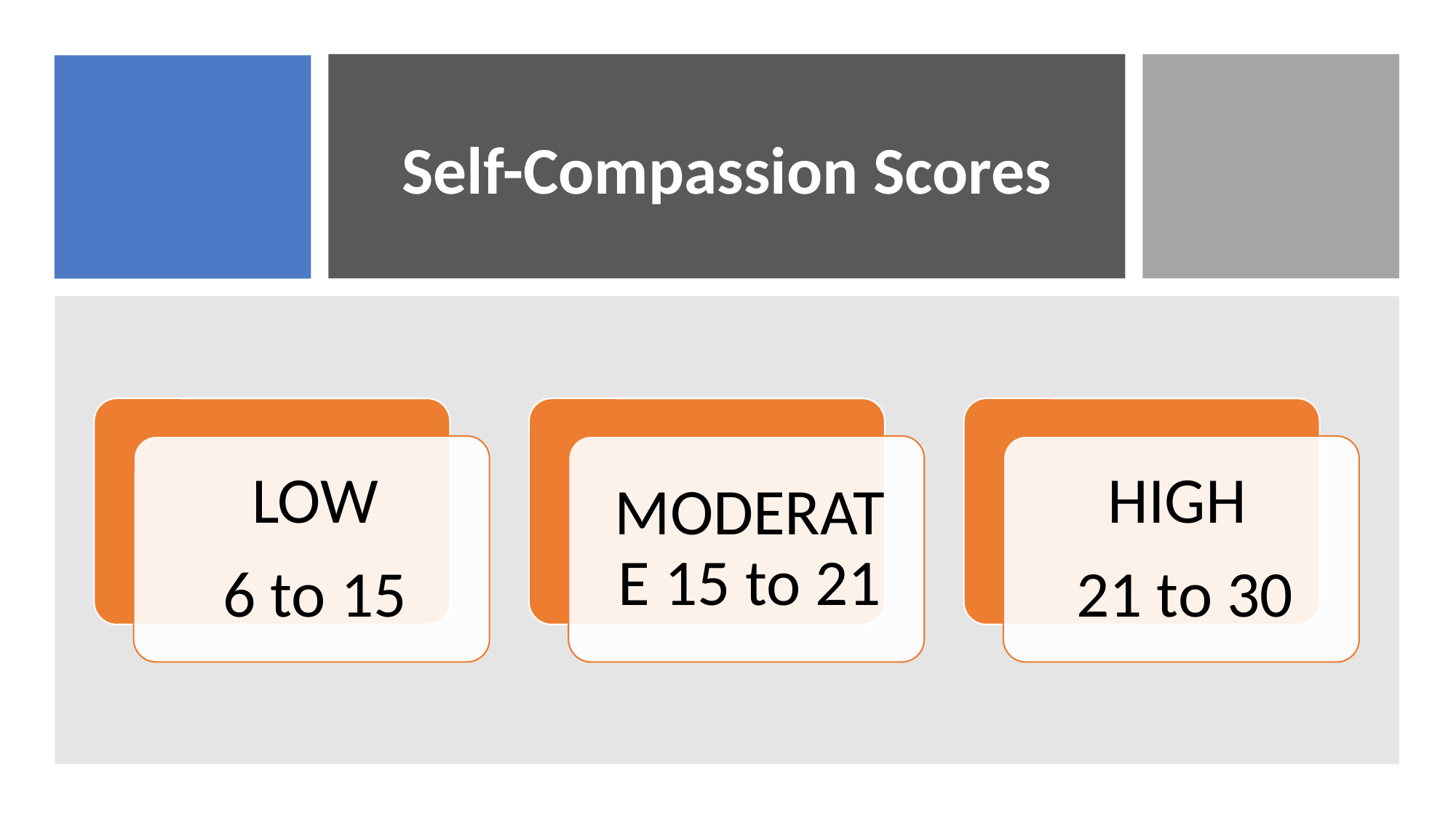

# Self-Compassion Scores

## Slide 14
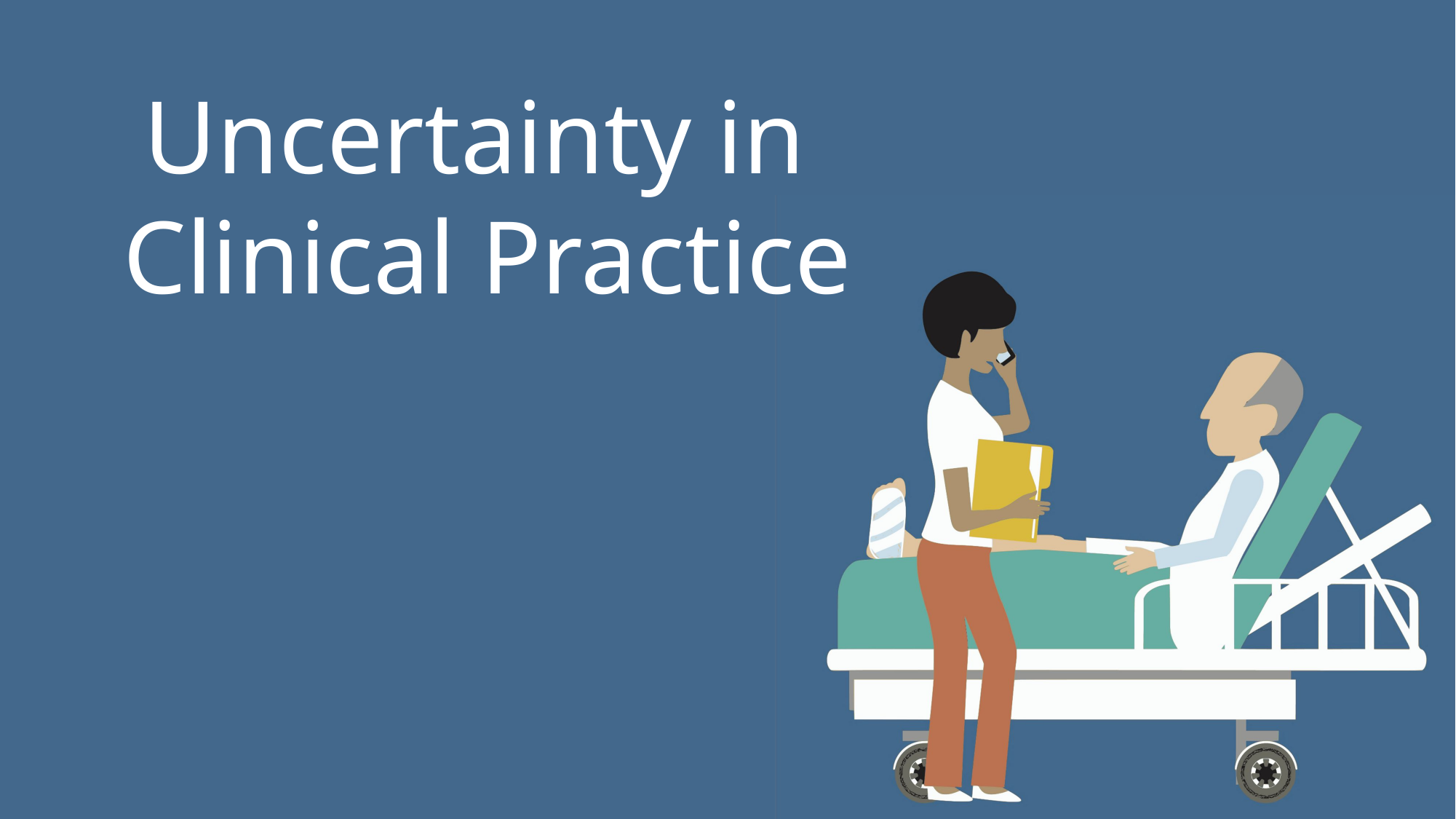

Uncertainty in
Clinical Practice

## Slide 15
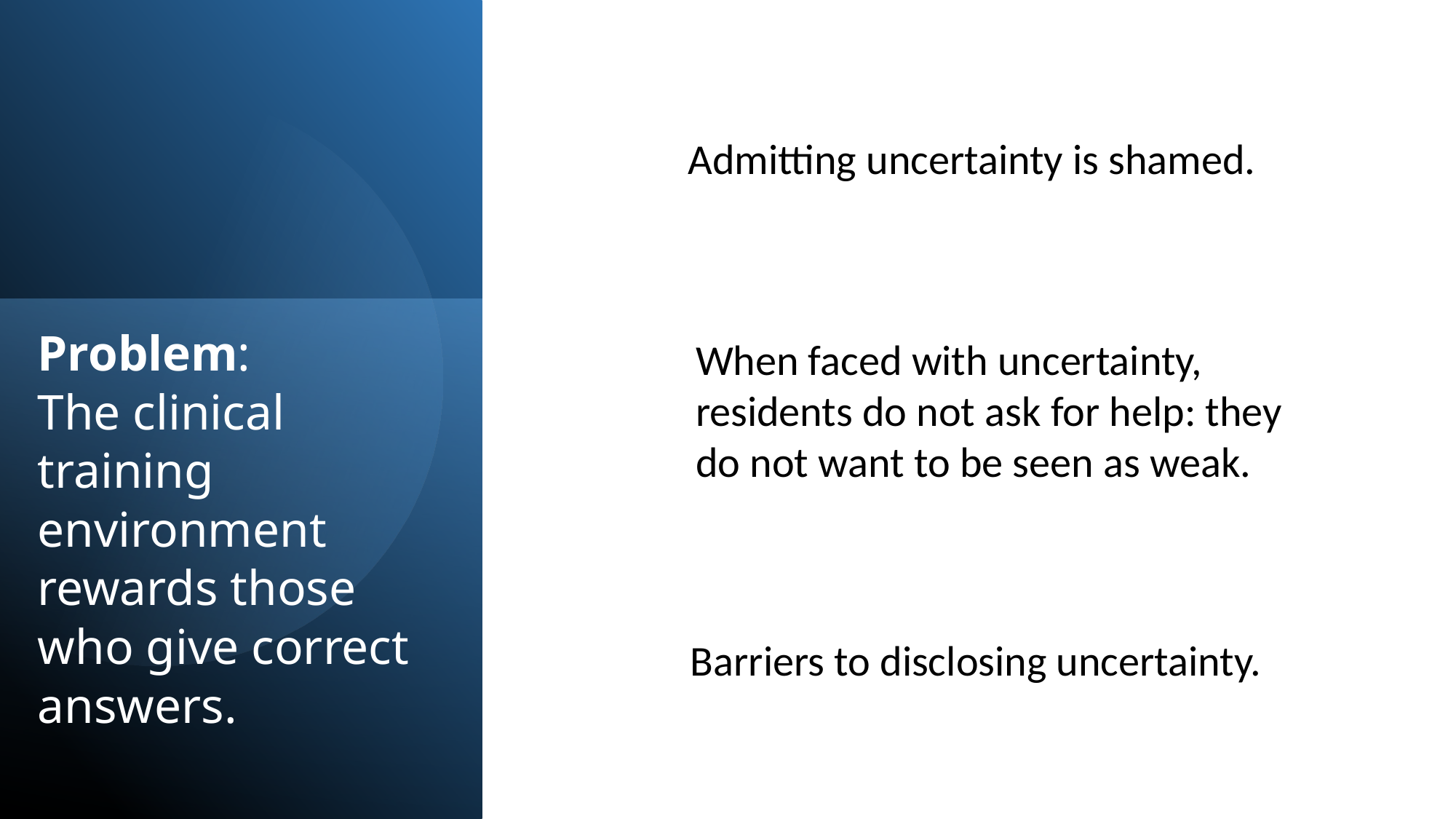

Admitting uncertainty is shamed.
When faced with uncertainty, residents do not ask for help: they do not want to be seen as weak.
# Problem: The clinical training environment rewards those who give correct answers.
Barriers to disclosing uncertainty.

## Slide 16
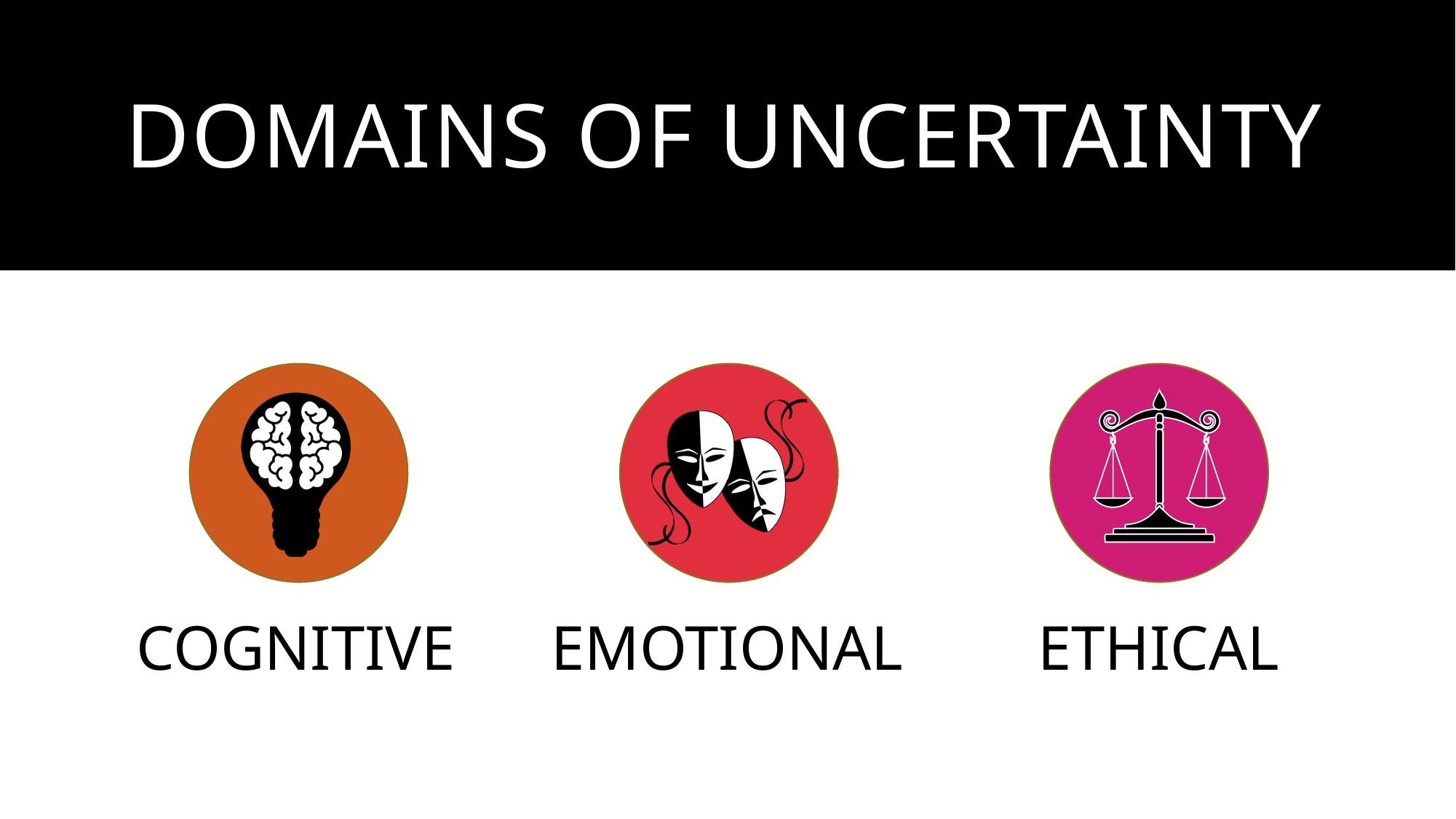

# Domains of Uncertainty
Emotional
Cognitive
Ethical

## Slide 17
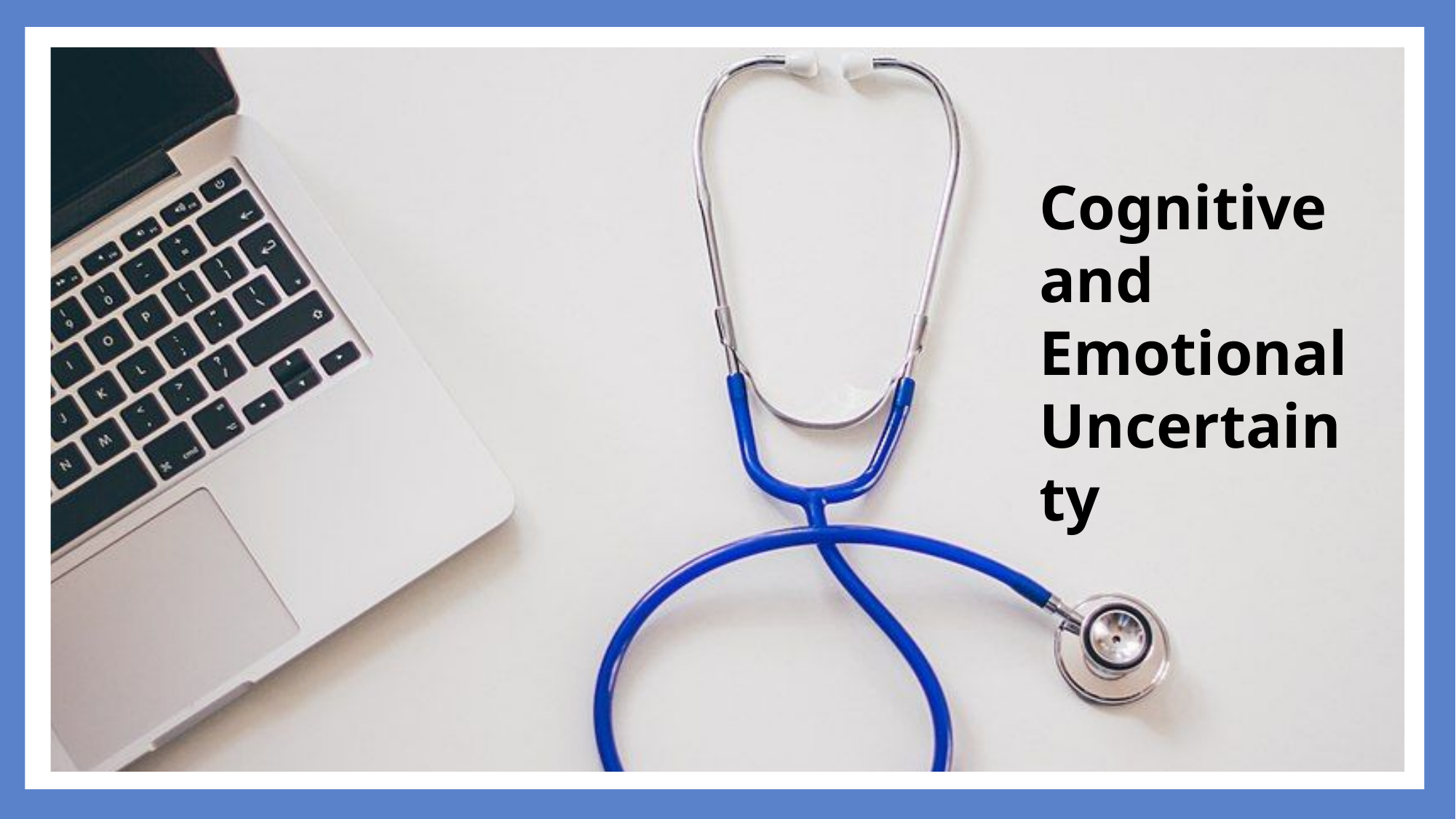

Cognitive and Emotional Uncertainty

## Slide 18
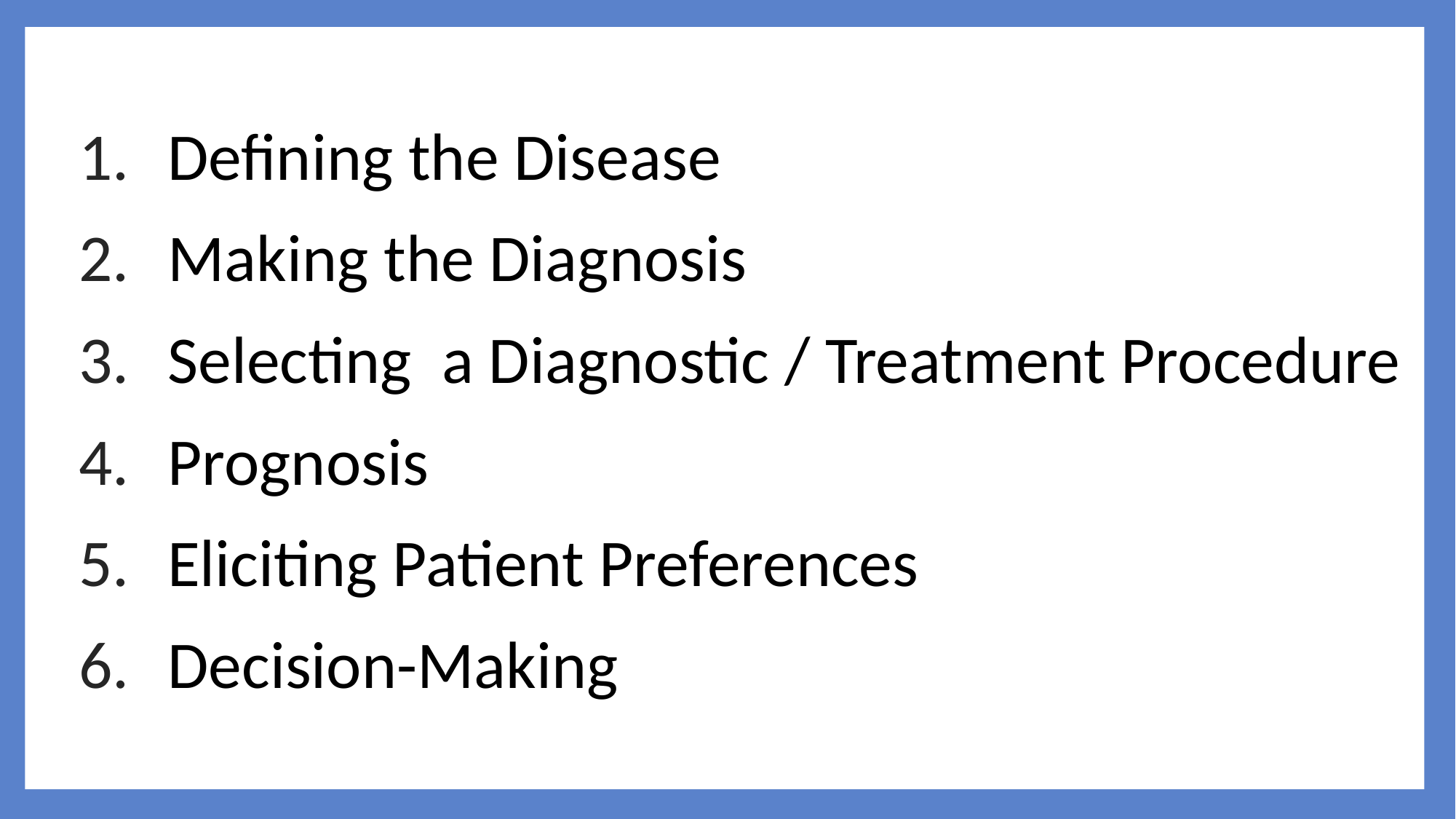

Defining the Disease
Making the Diagnosis
Selecting a Diagnostic / Treatment Procedure
Prognosis
Eliciting Patient Preferences
Decision-Making

## Slide 19
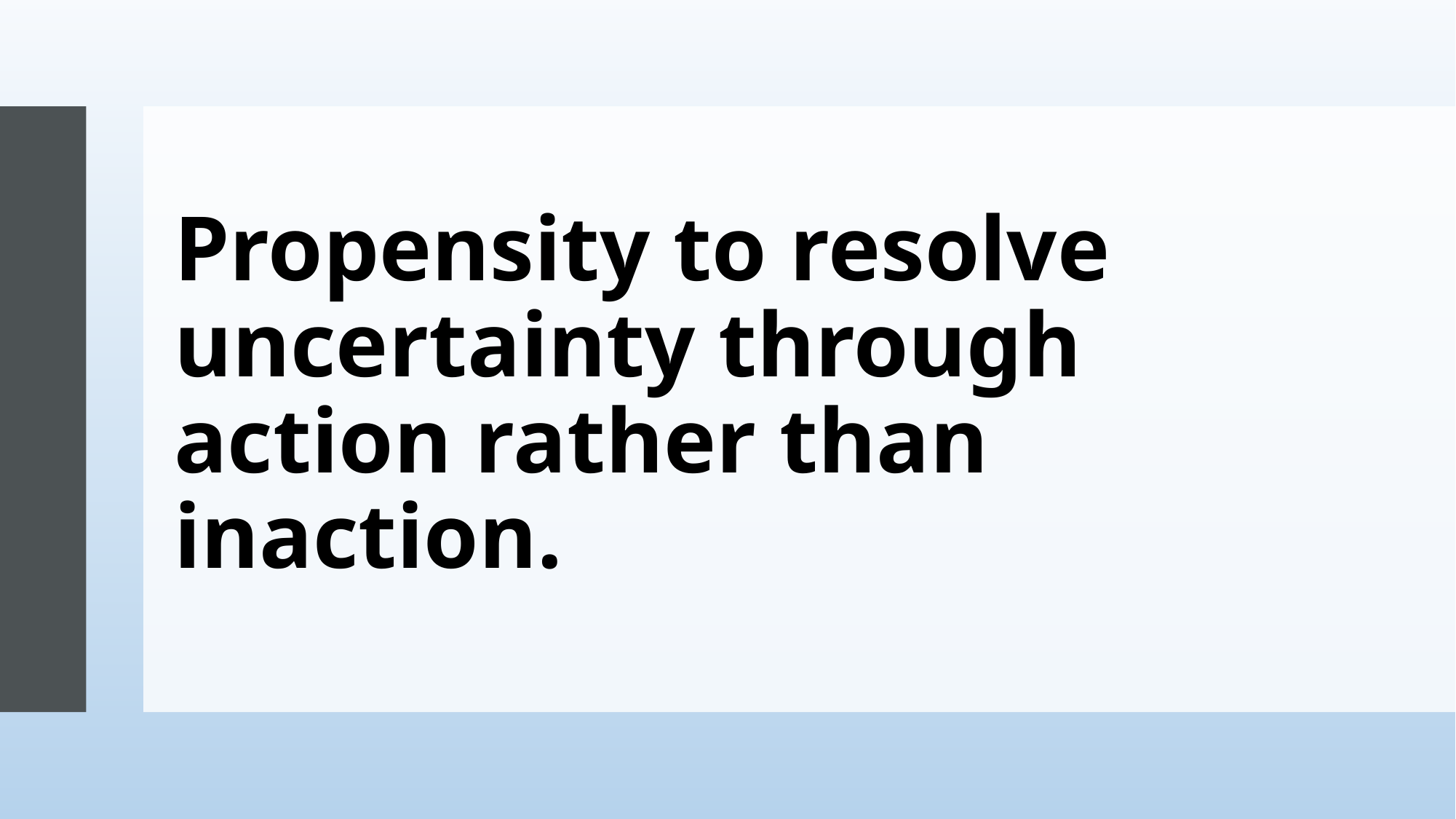

# Propensity to resolve uncertainty through action rather than inaction.

## Slide 20
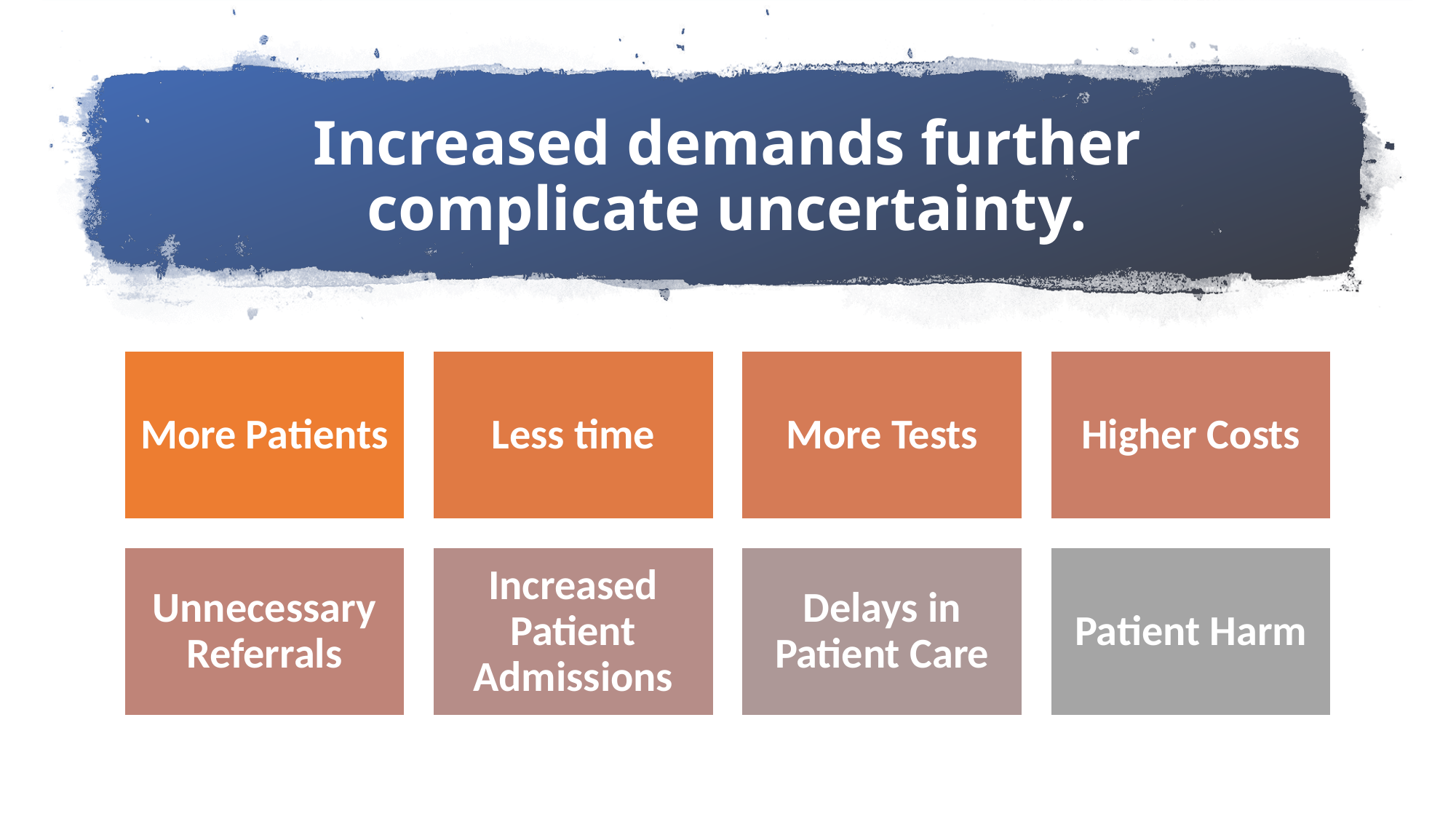

# Increased demands further complicate uncertainty.

## Slide 21
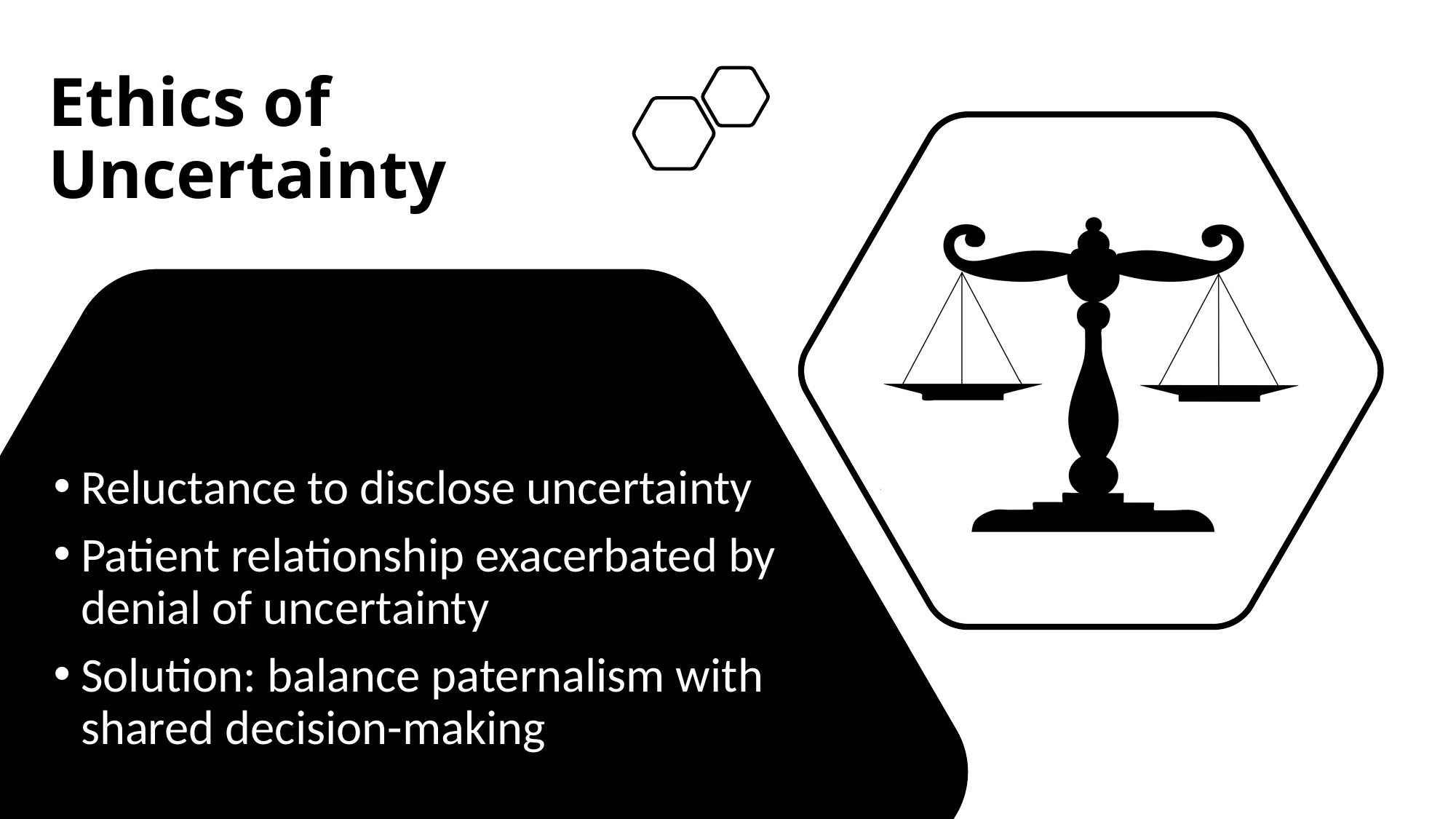

# Ethics of Uncertainty
Reluctance to disclose uncertainty
Patient relationship exacerbated by denial of uncertainty
Solution: balance paternalism with shared decision-making

## Slide 22
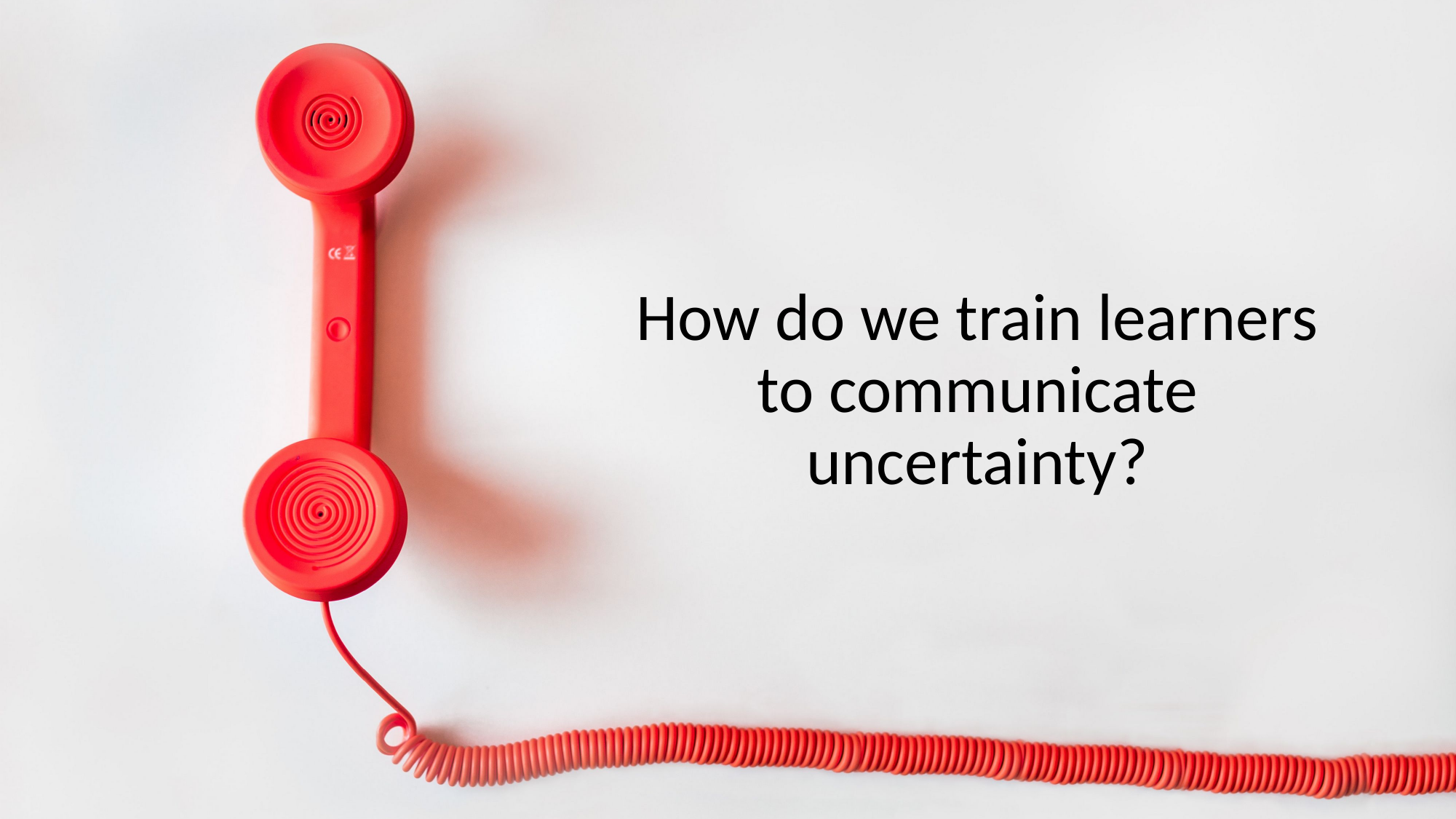

# How do we train learners to communicate uncertainty?

## Slide 23
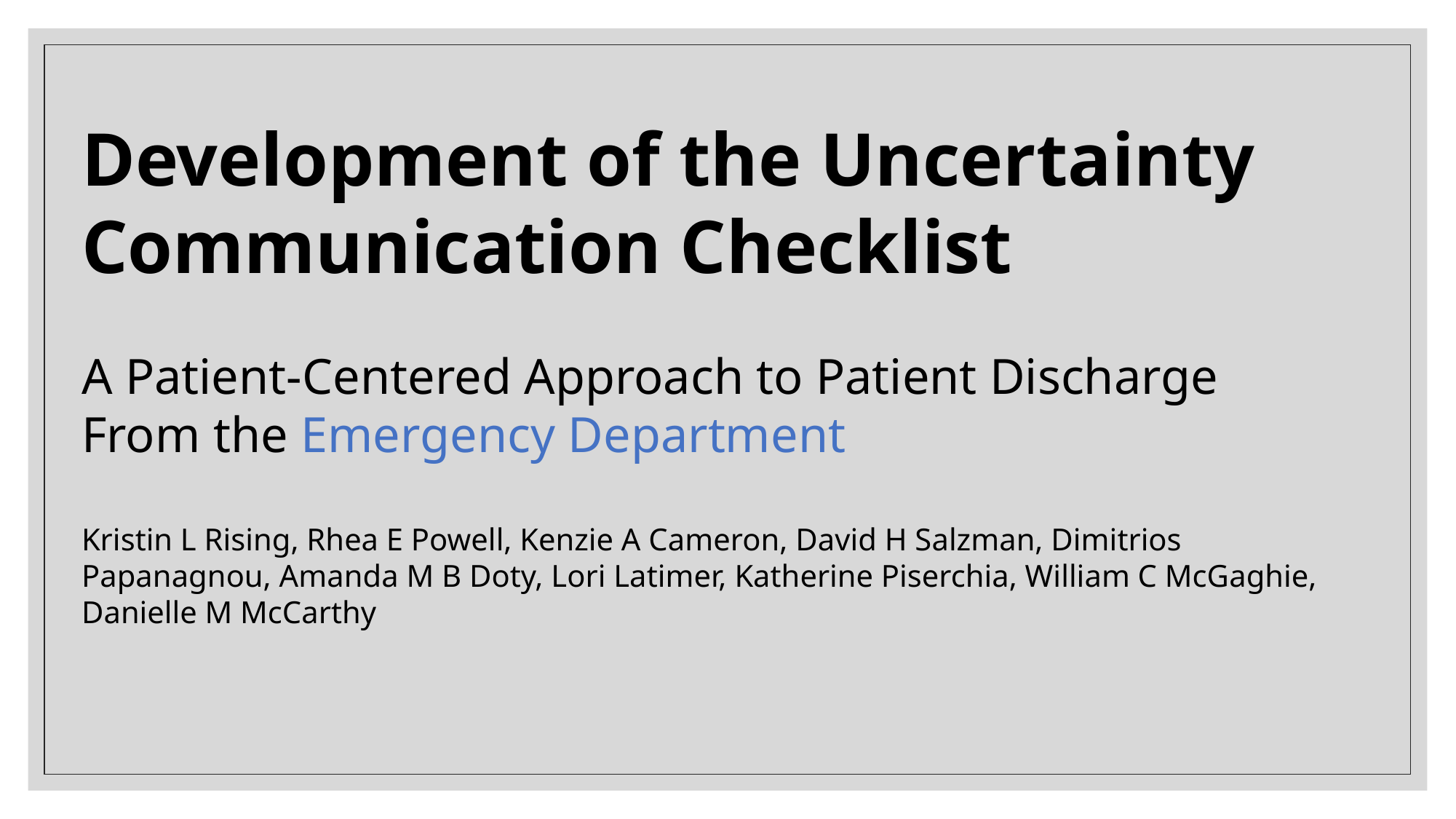

Development of the Uncertainty Communication Checklist
A Patient-Centered Approach to Patient Discharge From the Emergency Department
Kristin L Rising, Rhea E Powell, Kenzie A Cameron, David H Salzman, Dimitrios Papanagnou, Amanda M B Doty, Lori Latimer, Katherine Piserchia, William C McGaghie, Danielle M McCarthy

## Slide 24
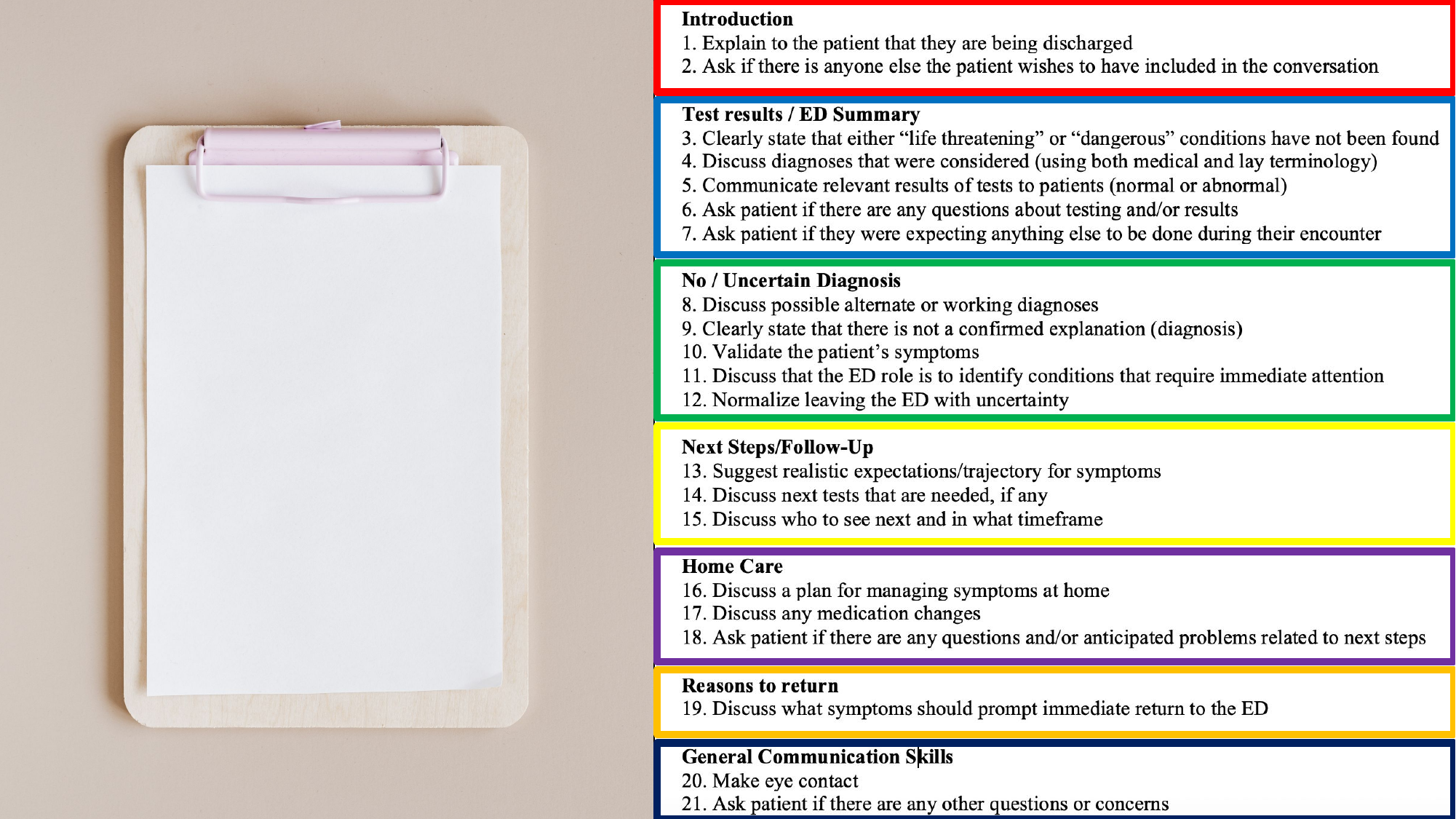

## Slide 25
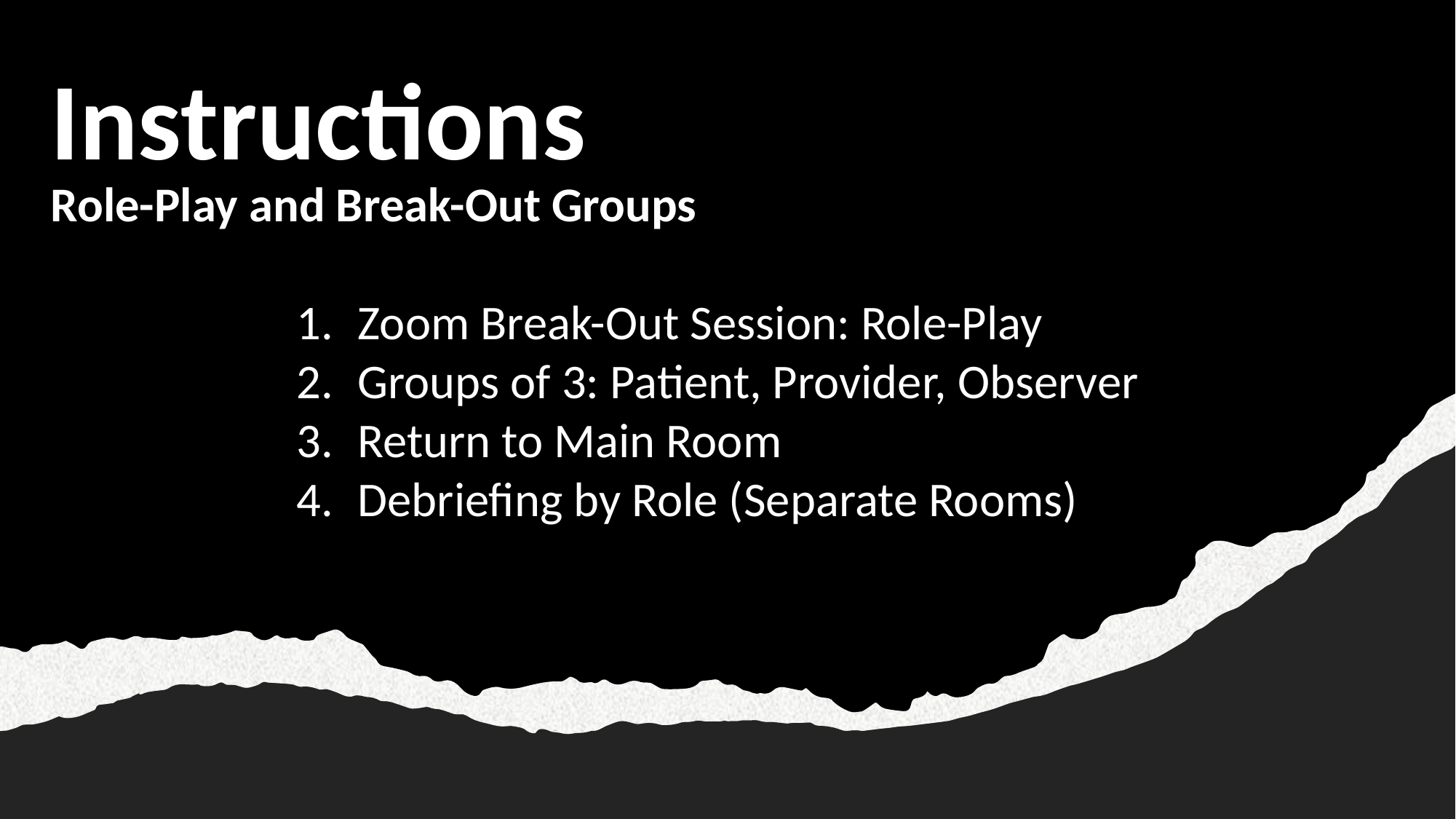

# Instructions
Role-Play and Break-Out Groups
Zoom Break-Out Session: Role-Play
Groups of 3: Patient, Provider, Observer
Return to Main Room
Debriefing by Role (Separate Rooms)

## Slide 26
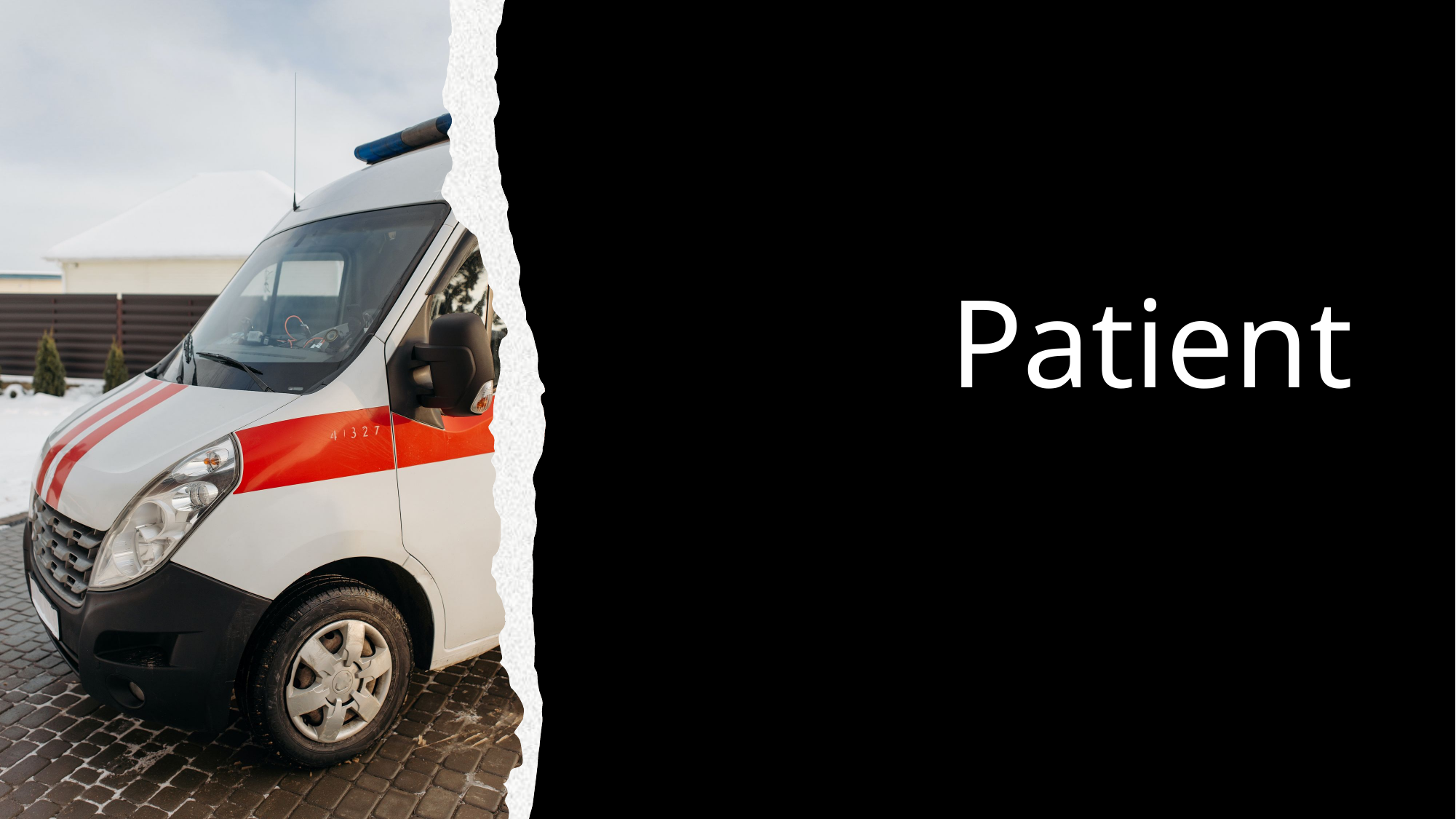

# Patient

## Slide 27
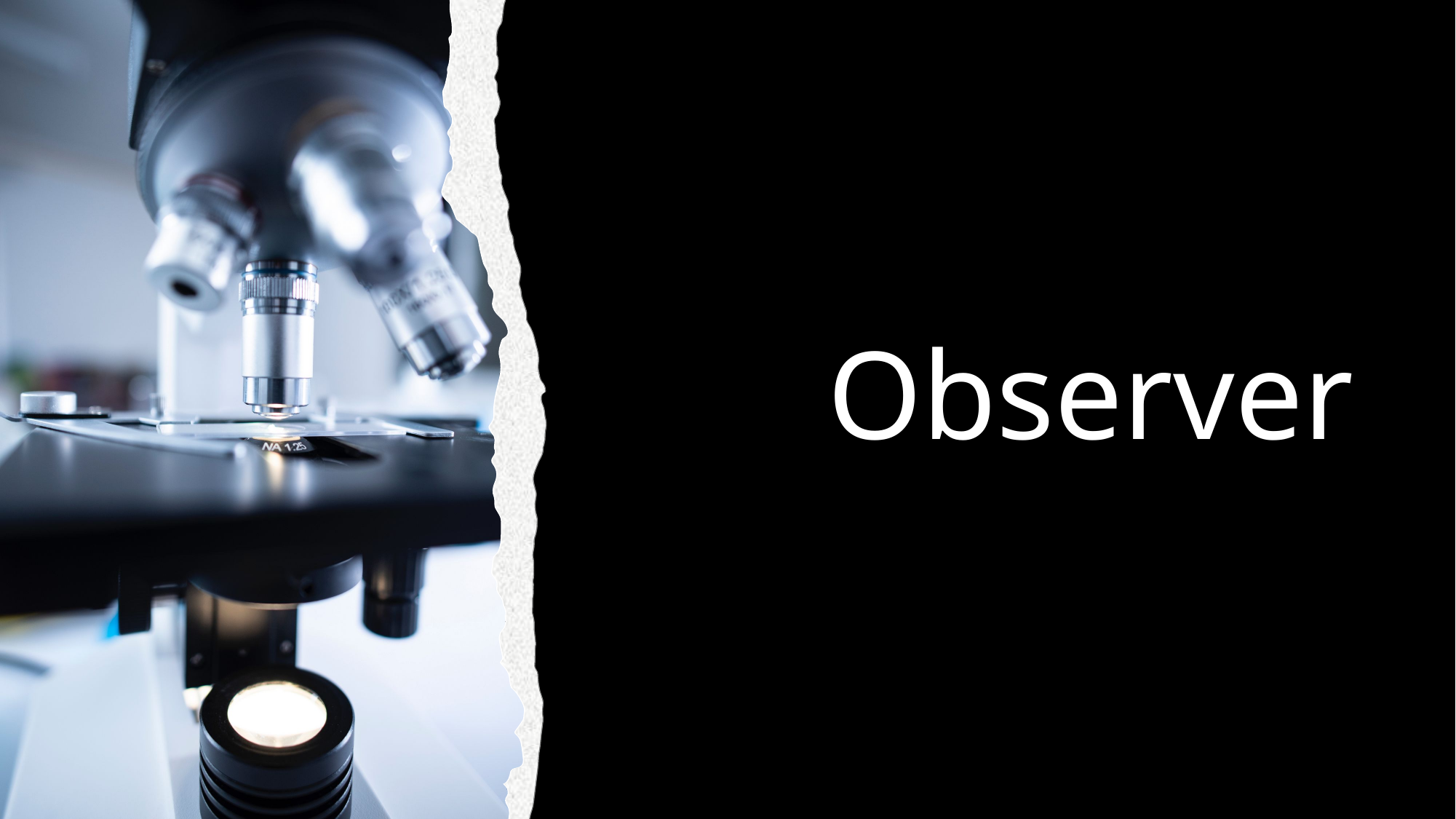

# Observer

## Slide 28
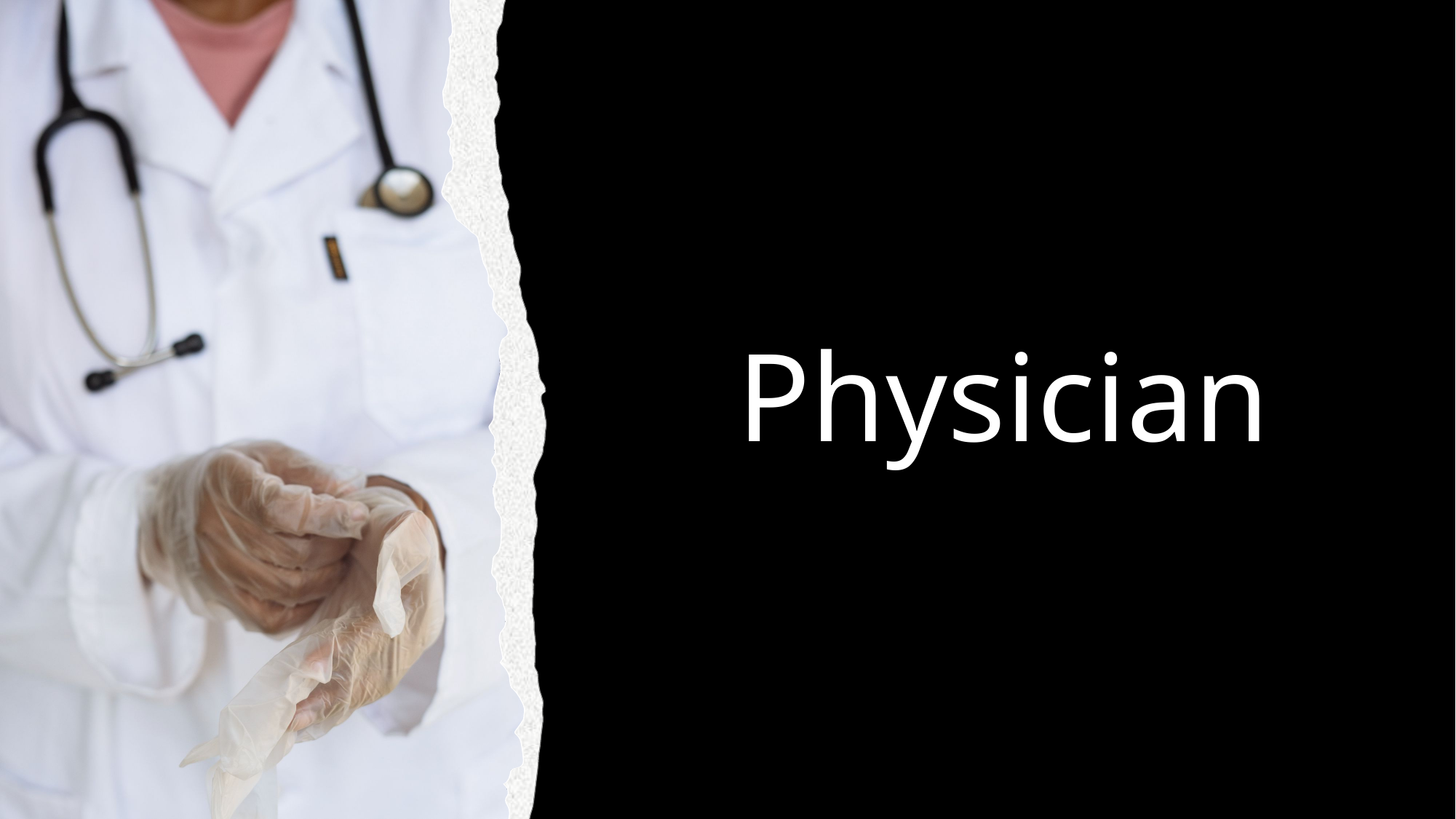

# Physician
