# Supplementary material for: I Don't Have a Diagnosis for You: Preparing Medical Students to Communicate Diagnostic Uncertainty in the Emergency Department
Source: MedEdPORTAL. 2022 Feb 4;18:11218. doi: 10.15766/mep_2374-8265.11218 (PMC8814030; doi:10.15766/mep_2374-8265.11218)
Supplement: Supplementary file 1 — Uncertainty Communication Checklist.docxPrework Reflection Prompts.docxIntolerance of Uncertainty Scale.docxSelf-Compassion Scale Short Form.pdfUncertainty Articulate Module folderDebrief Facilitator Prompts.docxCommunicating Diagnostic Uncertainty Slides.pptxSimulation Student Role-Play Instructions.docxPostsession Survey.docx [file mep_2374-8265.11218-s001.zip › H. Simulation Student Role-Play Instructions.docx]

**Instructions for Student Playing the Role of the Physician**

**Patient’s Name:** Patient Peterson

**Age:** 55-year-old patient

**Setting:** Emergency Department

**Initial Chief Complaint on arrival to the ED:** Abdominal pain

**Vital Signs over Course of Evaluation:**

HR 64 bpm; BP 135/78 mmHg; RR 18; 100% RA; 98.8 F (oral).

VS stable during ED course.

**Patient Information:**

55-year-old patient with no PMH, who presented to the ED with 1-day history of left lower quadrant pain (LLQ) pain. Patient arrived to the ED with normal vital signs, afebrile, with reproducible tenderness in the LLQ. The remainder of the remainder of exam was unremarkable. Initial plan was to check labs and a urinalysis, and to obtain a CT scan to evaluate for possible diverticulitis versus any other occult intra-abdominal pathology. Patient was given Tylenol, which improved the pain, as well as one liter of normal saline.

**Results:** Labs and urinalysis were unremarkable. Impression of CT results (see below) are normal.

**ED Course:**

Most recent vitals: HR 68 bpm; BP 130/74 mmHg; RR 16; 100% RA; 98.8 F (oral).

Repeat exam: no tenderness in the LLQ

**Your Task:**

- Approach the patient and disclose the results, including the CT scan.
- Should the patient have any concerns with these results, discuss them with the patient.
- Discharge the patient from the Emergency Department.

**Results from the CT scan of the abdomen and pelvis:**

CT Abd/pelvis:

Type of exam: Computed tomography (CT) of the abdomen and pelvis with intravenous and oral contrast.

Clinical history: 55-year-old with new onset of left lower quadrant pain. Concern for diverticulitis.

Comparison: No previous imaging for comparison.

Technique: 5-mm axial images from the lung bases through the pubic symphysis were acquired following the administration of intravenous and oral contrast. Coronal and sagittal reformatted images were constructed from source data.

Findings:

Lung bases: No pulmonary nodules or evidence of pneumonia.
Cardiac: Base of heart is within normal limits. No pericardial effusion.
Liver: Normal size and contour.

Gallbladder: Normal appearance, no gallstones.
Biliary: No intra or extrahepatic biliary dilation.
Spleen: No splenomegaly.
Pancreas: No mass or ductal dilation.
Kidneys and Adrenals: No masses, stones or hydronephrosis. No adrenal nodules.
Lymph nodes: No lymphadenopathy.
Bowel: No dilation or wall thickening.
Bladder: Within normal limits.
Uterus and Adnexa: The uterus and bilateral ovaries are within normal limits for age.
Bones and soft tissue: There are no osseous or soft tissue abnormalities.
Other: No free fluid within the pelvis.

Impression:

Normal CT of the abdomen and pelvis. No findings on the current CT to explain the patient’s clinical presentation of abdominal pain.

**Instructions for Student Playing the Role of the Patient**

**Patient Name:** Patient Peterson

**Age Range:** 55 y/o

**Setting:** Emergency Department

**Initial Presenting Symptoms:** Left Lower Quadrant Abdominal Pain

**Your Emotional State:** Nervous and Anxious

To better understand the emotional state you are playing, please see below:

| **Your emotional state in the Emergency Department can be described as nervous and anxious:** | Nervous: You were scared to come to the ED for evaluation because your family member recently received a cancer diagnosis in the ED and it took you a lot of courage to come in for evaluation of your abdominal pain.  Anxious: You are worried and nervous about your CT results. A friend of yours was recently diagnosed with colon cancer, and you are terrified about this potential diagnosis. You still do not know the results of the CT, which has been performed hours ago. You fear that the delay may be because of bad news. |
| --- | --- |

**Clinical Background to Better Understand Your Role:**

You are a 55-year-old patient with no significant past medical history who presented to the ED for the evaluation of abdominal pain, specifically in your left lower quadrant (LLQ), which started one day ago. The pain is intermittent with no radiation. It is dull in quality. You have not traveled or taken antibiotics. You have no PSH. You have never experienced pain like this in the past. You have no complaints urinating. Your bowel movements are normal.

After a lengthy wait in the ED, you were seen by the treating team; your labs and urine studies were ordered; and you underwent a CT scan with IV and PO contrast media.

It has been 2 hours since your CT, and no one has updated you about your results. You are nervous (as your friend was recently diagnosed with colon cancer). You want to know what is causing your pain. You want a diagnosis. You want peace of mind that this is not cancer.

**What To Do During the Role-Play:**

- Greet the physician:
  - “*Hello, doctor. I hope you have some news for me. I have been waiting since the morning. I have been here for hours. I am tired and I am hungry. I’ve had my CT performed, and no one has updated me with the results. Can you please tell me what’s going on!?*”
- Express nervousness with regards to the delays.
- Share your anxiety with ‘negative’ results when they are disclosed to you.
- Ask the physician to tell you what he/she sees on the CT scan that can explain your abdominal pain.
- Express anxiety and nervousness when the physician informs you that the CT scan does not explain the cause of your abdominal pain.
- Ask the physician: “*So, what is wrong with me?*”
  - State that you are “nervous” when there is no diagnosis disclosed.
  - Question how there can be “no diagnosis.”

**Instructions for Student Playing the Role of the Observer**

- During the encounter, you will watch a physician discharge a patient from the Emergency Department (ED).
- The patient presented to the ED with abdominal pain. After an extensive course, tests, and a CT scan, results are all within normal limits. The physician will discharge the patient home without a diagnosis.
- Pay close attention to the conversation between the student physician and the student patient.
- Please use the checklist (below) to see if any of these important elements are covered in the conversation. You will use these observations to provide feedback to your peers.

**Uncertainty Communication Checklist for Patient Discharge From the Emergency Department**

|  | **Introduction**  1. Explain to the patient that they are being discharged  2. Ask if there is anyone else whom the patient wishes to have included in the conversation in person and/or by phone |
| --- | --- |
|  | **Test results/ED summary**  3. Clearly state that either “life-threatening” or “dangerous” conditions have not been found  4. Discuss diagnoses that were considered (using both medical and lay terminology)  5. Communicate relevant results of tests to patients (normal or abnormal)  6. Ask patient if there are any questions about testing and/or results  7. Ask patient if they were expecting anything else to be done during their encounter—if yes, address reasons not done |
|  | **No/uncertain diagnosis**  8. Discuss possible alternate or working diagnoses  9. Clearly state that there is not a confirmed explanation (diagnosis) for what the patient has been experiencing  10. Validate the patient’s symptoms  11. Discuss that the ED role is to identify conditions that require immediate attention  12. Normalize leaving the ED with uncertainty |
|  | **Next steps/follow-up**  13. Suggest realistic expectations/trajectory for symptoms  14. Discuss next tests that are needed, if any  15. Discuss who to see next and in what time frame |
|  | **Home care**  16. Discuss a plan for managing symptoms at home  17. Discuss any medication changes  18. Ask patient if there are any questions and/or anticipated problems related to next steps (selfcare and future medical care) after discharge |
|  | **Reasons to return**  19. Discuss what symptoms should prompt immediate return to the ED |
|  | **General communication skills**  20. Make eye contact  21. Ask patient if there are any other questions or concerns |
