# Supplementary material for: I Don't Have a Diagnosis for You: Preparing Medical Students to Communicate Diagnostic Uncertainty in the Emergency Department
Source: MedEdPORTAL. 2022 Feb 4;18:11218. doi: 10.15766/mep_2374-8265.11218 (PMC8814030; doi:10.15766/mep_2374-8265.11218)
Supplement: Supplementary file 1 — Uncertainty Communication Checklist.docxPrework Reflection Prompts.docxIntolerance of Uncertainty Scale.docxSelf-Compassion Scale Short Form.pdfUncertainty Articulate Module folderDebrief Facilitator Prompts.docxCommunicating Diagnostic Uncertainty Slides.pptxSimulation Student Role-Play Instructions.docxPostsession Survey.docx [file mep_2374-8265.11218-s001.zip › I. Postsession Survey.docx]

**Post-Session Survey**

**Q1 - Please indicate your comfort level with communicating diagnostic uncertainty with patients after today's workshop.**

Extremely comfortable

Somewhat comfortable

Neither comfortable or uncomfortable

Somewhat uncomfortable

Extremely uncomfortable

**Q2 – This is the first year we have included this workshop in the Gateway curriculum. Should this workshop be included in the Gateway curriculum in the future?**

Yes

No

**Q3 – Did you complete the Communicating Diagnostic Uncertainty Module prior to today’s workshop?**

Yes

No

**Q4 - How useful was the module in preparing you for today's workshop?**

Extremely useful

Very useful

Moderately useful

Slightly useful

Not at all useful

**Q5 – How useful was today’s workshop in preparing you to communicate diagnostic uncertainty with patients?**

Extremely useful

Very useful

Moderately useful

Slightly useful

Not at all useful

**Q6 - Did you complete any of the pre-reflection activities prior to attending today's workshop (e.g., the Intolerance of Uncertainty Scale, the Self-Compassion Scale, the Reflection Questions)?**

Yes

No

**Q7 – How useful was it reflecting on your views of uncertainty before today’s workshop?**

Extremely useful

Very useful

Moderately useful

Slightly useful

Not at all useful

**Q8 - What role did you specifically play during the workshop role-play?**

Role of patient

Role of physician

Role of observer

I did not participate in the role-play

**Q9 - Please offer any suggestions to improve this workshop in the future.**

**Q10 - Please share THREE ACTIONS discussed today that you will apply when communicating with patients during times of diagnostic uncertainty.**
